# Structural Features of DNA in TATA-Containing and TATA-Less Core Promoters of RNA Polymerase II Differ

**DOI:** 10.3390/ijms27177735

**Published:** 2026-08-28

**Authors:** Ekaterina A. Savina, Olga D. Soldatenkova, Anastasia A. Anashkina, Irina A. Il’icheva

**Affiliations:** 1 Engelhardt Institute of Molecular Biology, Russian Academy of Sciences, Vavilova 32, Moscow 119991, Russia; e.savina6118@gmail.com (E.A.S.);; 2 Department of Information and Internet Technologies, Institute of Digital Biodesign and Artificial Intelligence in Medicine, Sechenov University, 8-2 Trubetskaya Str., Moscow 119991, Russia

**Keywords:** eukaryotes, transcription apparatus, core promoter DNA local structure, ultrasonic cleavage, DNase I cleavage, TATA box, TATA-less

## Abstract

Nucleotide motifs in the core promoters of eukaryotic protein-coding genes transcribed by RNA polymerase II (Pol II) play an important role in the transcription process. We analyzed the role of an octanucleotide located in the TATA box position. Depending on whether this octanucleotide can form a complex with the TATA-binding protein (TBP), the promoter is classified as either TATA-containing or TATA-less. We analyzed the differences in the primary and spatial structures, as well as their dynamics, in TATA-containing and TATA-less promoters of mammals and plants. We divided the complete promoter sets of six organisms (*H. sapiens*, *M. musculus*, *C. familiaris*, *A. thaliana*, *Z. mays*, and *H. vulgare*) from the EPDnew database into TATA-containing and TATA-less fractions. The sizes of the TATA-containing promoter fractions are significantly smaller than those of the TATA-less fractions in all studied organisms, except in *A. thaliana*, where the sizes of both fractions are approximately equal. We characterized promoter architecture using variation profiles of various base-pair step parameters, minor-groove width, and the conformational dynamics of native DNA. The architectures of TATA-containing and TATA-less promoters differ significantly. The possible mechanistic influence of DNA structural features on the formation of the pre-initiation complex (PIC) in both types of promoters is discussed.

## 1. Introduction

The high complexity of protein-coding gene transcription by Pol II, particularly PIC formation, which occurs in core promoters [1,2,3], underscores the importance of a detailed analysis of the primary and spatial structures, as well as the dynamics, of double-stranded DNA in this region. The core promoter of protein-coding genes is typically located in the −50 to +50 bp region around the transcription start site (TSS) and controls the initial stages of transcription. The primary structure of these promoters contains a variety of motifs, and their specific sets vary depending on the promoter type [4].

We have previously examined this region of promoters of 15 organisms, namely ten animals (vertebrates, invertebrates, and insects)—*H. sapiens*, *M. mulatta*, *M. musculus*, *R. norvegicus*, *C. familiaris*, *G. gallus*, *D. rerio*, *C. elegans*, *D. melanogaster*, and *A. mellifera*; two plants—*A. thaliana* and *Z. mays*; two unicellular fungi—*S. cerevisiae* and *S. pombe*; and one protozoan—*P. falciparum*. We discovered a characteristic structure in this region. It displays a distinct landscape of changes in spatial conformational parameters and conformational dynamics [5,6]. In addition, we obtained sets of octanucleotides located at the TATA box, typically at positions −30 to −23 in mammalian promoters or −34 to −27 in plants. The most frequently occurring octanucleotides specific to each organism are presented in Table 1 in [6]. It is important to note that their frequency of occurrence ranges from 1% to 10^−2^%. Consequently, in each of the 15 organisms, the TATA box is represented by more than a hundred variants of octanucleotides, completely different not only in sequence but also in structural properties. Octanucleotides fitting the TATAWAWR consensus account for only a small fraction of the 20 most frequently occurring variants.

The motif located within the TATA box plays a crucial role in the transcription process, largely determining its efficiency [4]. A systematic X-ray crystallographic study of a canonical wild-type TBP (*A. thaliana* TBP isoform 2) recognizing 10 naturally occurring variants of the AdMLP TATA element was performed in [7], and it was shown that “TBP can bind productively to a large number of diverse TATA elements, some of which bear little resemblance to the optimal TATA sequence”. Moreover, the structure of the TBP–DNA complex is essentially independent of TATA-element sequence, while the affinity of TBP for the octanucleotide depends on both its sequence and structural properties [7].

Data from the ENCODE project indicate that promoters with the TATAWAWR consensus represent a minority of mammalian promoters in mice and humans [8]. This class of promoters is commonly associated with tissue-specific genes.

Previously, several properties of TATA-containing and TATA-less promoters were compared within the −500 to +500 bp region around the TSS [9]. In that study, promoters were separated into two fractions based on the presence of the TATAWAWR consensus. Significant differences were observed in DNA duplex stability, bendability, and curvature as well as in the distribution of sequence motifs, such as A-tracts, G-quadruplexes, and CpG islands, in TATA-containing and TATA-less promoters in mice and humans. However, because the analysis was performed on such a broad region, the fine structural details of the core promoter near the TSS were not considered.

In this study, we focused specifically on the −50 to +30 bp fragment of core promoters, which includes two key regions: the initiator (Inr) hexanucleotide around the TSS and the octanucleotide within the TATA box, located approximately −28 bp upstream. This fragment also contains various motifs that play different roles in PIC formation. It exhibits a distinct landscape of spatial architecture and conformational dynamics [5,6].

If we do not take into account the efficiency of binding of the octanucleotide to TBP, then we can assume that the TATA box can contain one of two types of octanucleotides: one that can form a complex with TBP, and one that cannot. We classified a promoter to the TATA-containing type if an octanucleotide at the TATA position is potentially capable of forming a complex with TBP in accordance with the sequence restriction rules from [7] (although their binding affinity may vary); otherwise, we will refer to it as TATA-less.

Our goal was to reveal the specific features of TATA-containing promoters and their differences from TATA-less promoters. Therefore, this work is devoted to the analysis of the primary and spatial structures, as well as conformational dynamics, in TATA-containing and TATA-less core promoters of mammals and plants.

## 2. Results

We retrieved promoter sets from the EPDnew section of the Eukaryotic Promoter Database (EPD; http://epd.vital-it.ch, accessed on 20 April 2026) [10]. This resource provides a collection of experimentally validated promoters for several model organisms. Transcription start site (TSS) mapping in this database was derived from high-throughput experiments such as CAGE and Oligo-capping, ensuring high precision and coverage.

We divided the complete promoter sets for *H. sapiens*, *M. musculus*, *C. familiaris*, *A. thaliana*, *Z. mays*, and *H. vulgare* into two fractions: TATA-containing and TATA-less depending on whether the octanucleotide in the TATA box can form a complex with TBP or not. To identify TATA-containing promoters, we applied a structure-based definition derived from high-resolution co-crystal structures of TBP with 10 natural TATA-element variants [7]; see details in the Materials and Methods Section.

According to these data, the minor-groove recognition surface of TBP imposes strict base-pair requirements at specific positions within the octanucleotide. The allowed nucleotides at positions 2, 4, and 5 are only A or T; C and G are strictly excluded due to steric clashes with conserved protein side chains (Leu-163 at position 2, Val-119 at position 4, and Val-29 at position 5). At position 3 A, T, or C are allowed; G is forbidden because its exocyclic NH_2_ group cannot be accommodated in the pocket formed by Val-119, Val-171, Thr-173 and Leu-163. At positions 1, 6, 7, and 8, all four nucleotides (A, T, G, C) are structurally compatible. At position 7, however, C or G require Hoogsteen base pairing to avoid a clash with Leu-72, which may reduce binding kinetics but does not preclude complex formation [7].

As a result of the splitting, we obtained a TATA-containing promoter fraction that was significantly smaller than the TATA-less fraction for all analyzed organisms, with the exception of *A. thaliana* (Table 1). Promoters in the TATA-containing fraction are highly heterogeneous in terms of their affinity for TBP, and even more so in terms of transcriptional activity. In [7], the 10 sequences representing naturally occurring single-base variants of the AdMLP TATA element (TATAAAAG), including at least one substitution at each position, were tested for transcription efficiency in vitro. A number of octanucleotides for which transcriptional activity was measured in [7] do not correspond to the TATAWAWR consensus, but have fairly high transcriptional activity compared to the TATATAAG transcription activity (TTTAAAAG activity is 20–29%, and AATAAAAG activity is 11–16%).

We compared the proportion of TATA-containing promoters with the proportion of promoters conforming to the TATAWAWR consensus. To do this, we compared the octanucleotides in the TATA box position with the TATAWAWR consensus. If the octanucleotide exactly conformed to the TATAWAWR consensus, we called it a promoter with the TATA consensus. Otherwise, it was called a TATA-non-consensus promoter. The split results are shown in Table 2.

The share of promoters containing the TATAWAWR consensus in the TATA box region is 3.3% (for *H. sapiens*)—5.9% (for *M. musculus* and *C. familiaris*) among TATA-containing promoters.

Thus, in the TATA box, octanucleotides with the consensus TATAWAWR are extremely rare. Moreover, data from [7] have shown in vitro that promoters whose TATA box does not meet the consensus, but can still bind to TBP, exhibit transcriptional activity. Therefore, in this study, we aimed to identify the structural features of TATA-containing promoters.

Consequently, all subsequent analyses of TATA-containing and TATA-less promoter characteristics were based on the classifications presented in Table 1.

### 2.1. Comparison of the Primary Structure of TATA-Containing and TATA-Less Promoters

The primary structures of the complete promoter sets and their fractions (TATA-containing and TATA-less) for each organism were characterized by nucleotide frequency profiles at each position across the −50 to +30 fragment, as well as by sequence logos.

**Figure 1:**
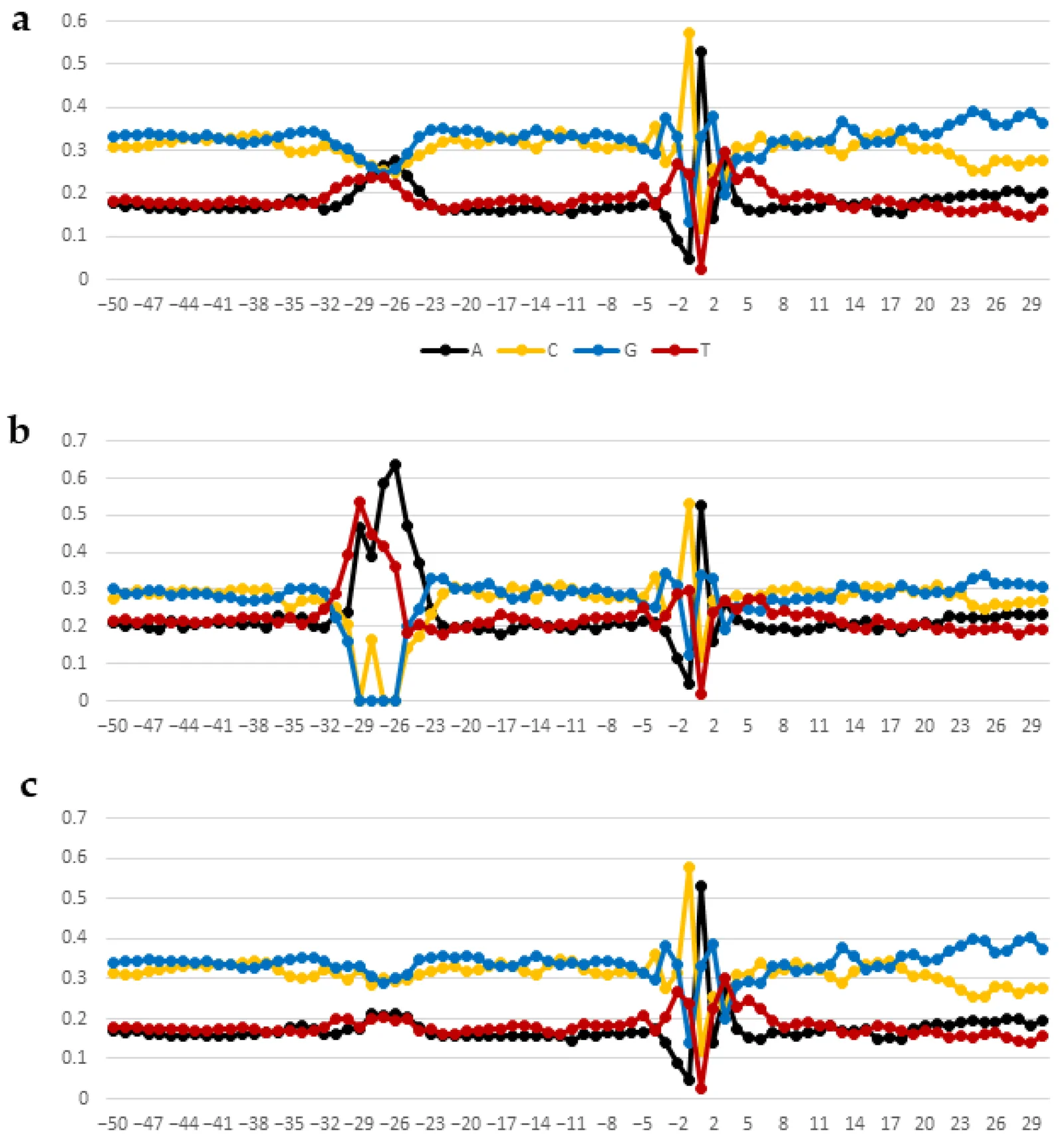
(**a**–**c**) Mononucleotide occurrence frequencies (in percentages) at each position along the coding strand of *H. sapiens*: (**a**) complete set of promoters; (**b**) TATA-containing promoters; (**c**) TATA-less promoters.

**Figure 2:**
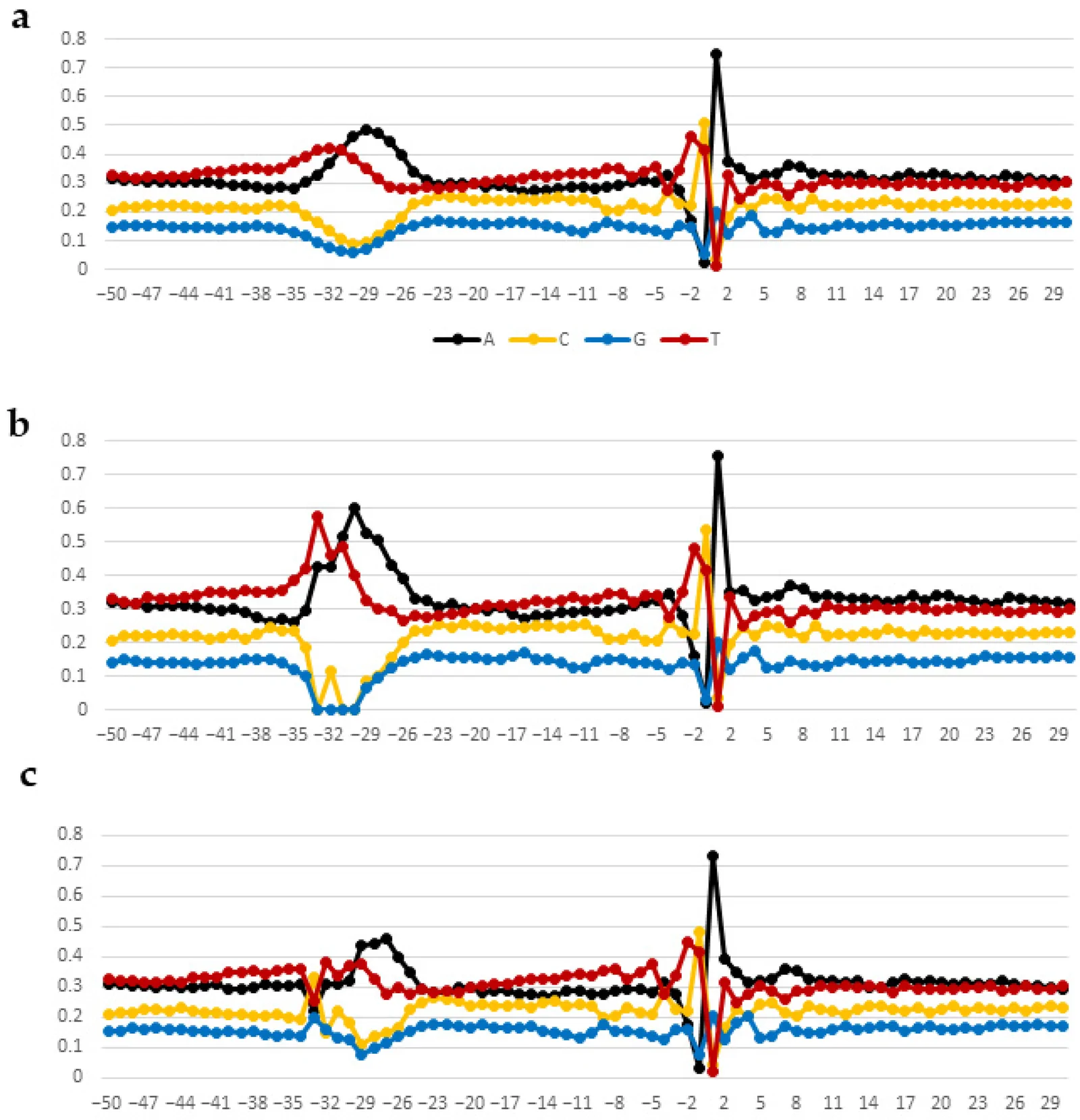
(**a**–**c**) Mononucleotide occurrence frequencies (in percentages) at each position along the coding strand of *A. thaliana*: (**a**) complete set of promoters; (**b**) TATA-containing promoters; (**c**) TATA-less promoters.

Nucleotide frequencies at each position are presented for *H. sapiens* (Figure 1a–c) and *A. thaliana* (Figure 2a–c). Analogous data for the other organisms are provided in the Supplementary Materials (Figure S1a–c for *M. musculus*, Figure S2a–c for *C. familiaris*, Figure S3a–c for *Z. mays*, and Figure S4a–c for *H. vulgare*).

Sequence logos with an information content of 1.0 bit for the complete promoter sets and both fractions of each organism are shown in the Supplementary Materials (Figure S5a–c for *H. sapiens*, Figure S6a–c for *M. musculus*, Figure S7a–c for *C. familiaris*, Figure S8a–c for *A. thaliana*, Figure S9a–c for *Z. mays*, and Figure S10a–c for *H. vulgare*).

**Figure 3:**
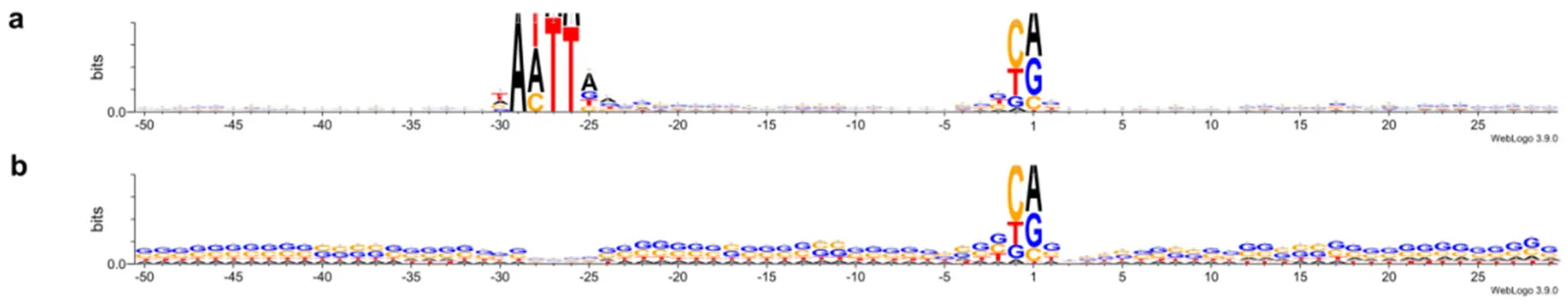
(**a**,**b**) LOGO representation with informational content 0.4 for TATA-containing (**a**) and TATA-less (**b**) promoters of *H. sapiens*.

**Figure 4:**
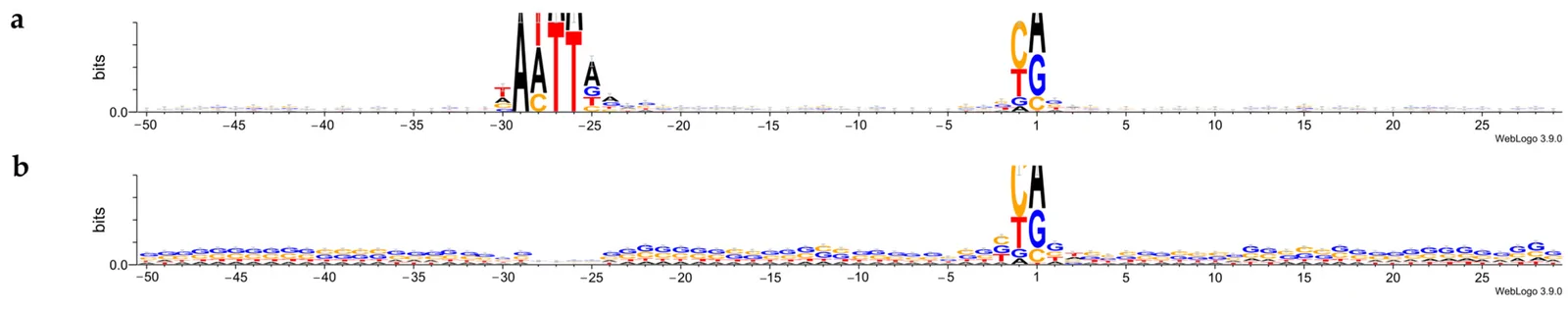
(**a**,**b**) LOGO representation with informational content 0.4 for TATA-containing (**a**) and TATA-less (**b**) promoters of *M. musculus*.

**Figure 5:**
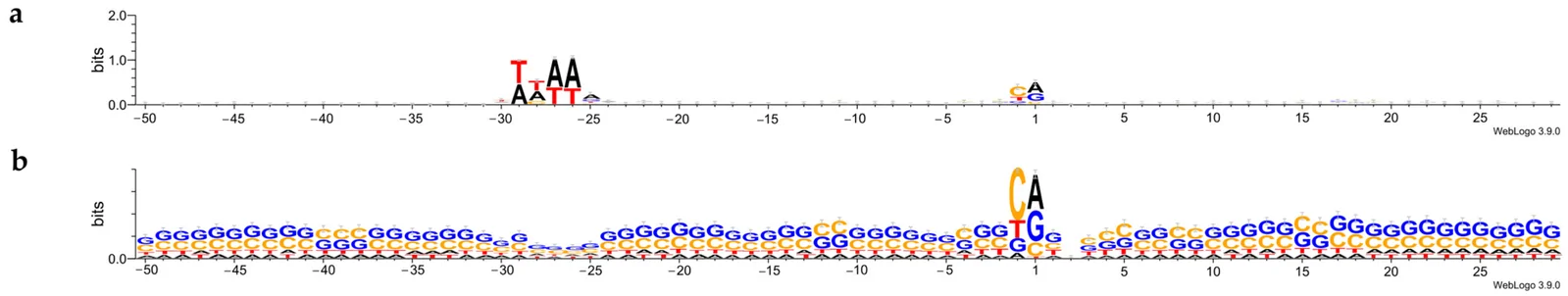
(**a**,**b**) LOGO representation with informational content 0.4 for TATA-containing (**a**) and TATA-less (**b**) promoters of *C. familiaris*.

**Figure 6:**
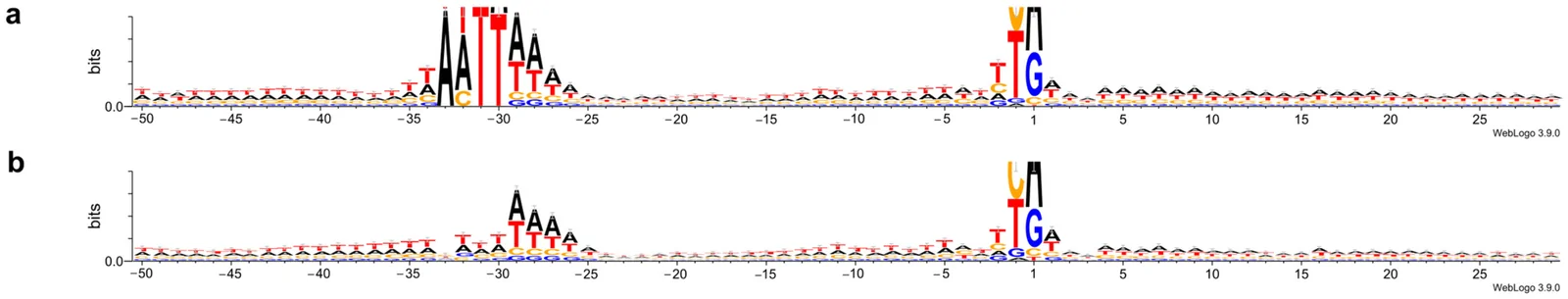
(**a**,**b**) LOGO representation with informational content 0.4 for TATA-containing (**a**) and TATA-less (**b**) promoters of *A. thaliana*.

**Figure 7:**
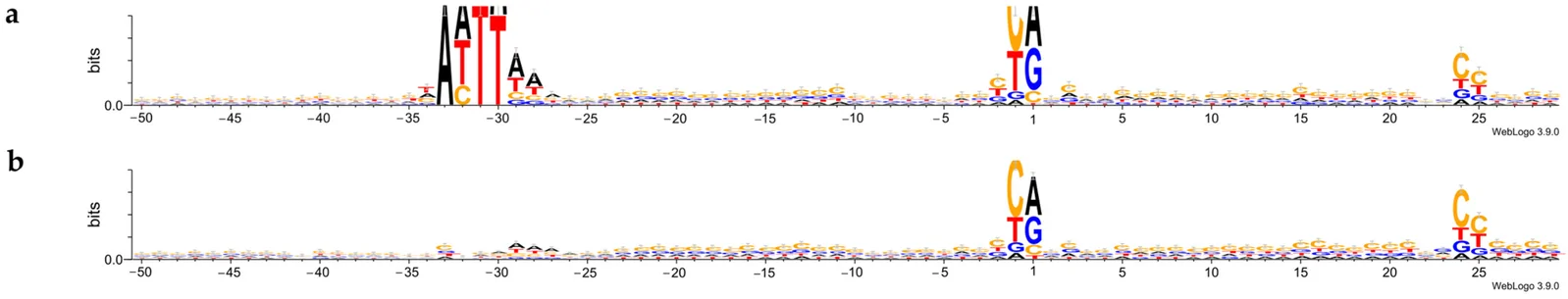
(**a**,**b**) LOGO representation with informational content 0.4 for TATA-containing (**a**) and TATA-less (**b**) promoters of *Z. mays*.

**Figure 8:**
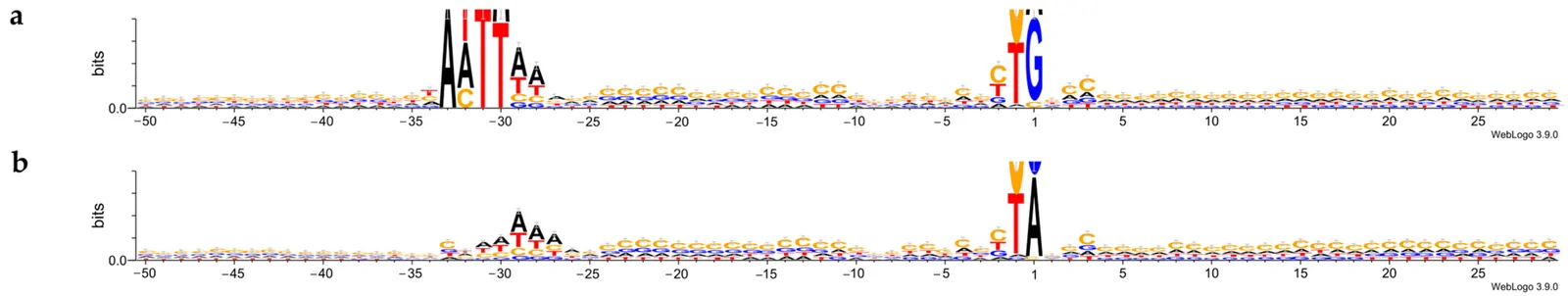
(**a**,**b**) LOGO representation with informational content 0.4 for TATA-containing (**a**) and TATA-less (**b**) promoters of *H. vulgare*.

Sequence logos with an information content of 0.4 bits for the TATA-containing and TATA-less fractions of each organism are shown in Figure 3a,b for *H. sapiens*, Figure 4a,b for *M. musculus*, Figure 5a,b for *C. familiaris*, Figure 6a,b for *A. thaliana*, Figure 7a,b for *Z. mays*, and Figure 8a,b for *H. vulgare*.

Significant differences are clearly visible between mammalian TATA-containing and TATA-less promoters. There is no predominance of TA in the TATA box of TATA-less promoters. However, in plants, TATA-less promoters preserve a slight predominance of TA in the TATA box (see Figure 3, Figure 4, Figure 5, Figure 6, Figure 7 and Figure 8)**.** The GC content around the TATA box is notably higher in TATA-less promoters than in TATA-containing promoters in all mammals, but not in plants (see Figure 3, Figure 4, Figure 5, Figure 6, Figure 7 and Figure 8).

### 2.2. Comparison of the Structural Properties of TATA-Containing and TATA-Less Promoters

The differences in the physical and structural properties of individual base-pair steps determine the spatial landscape of any DNA fragment. Therefore, to analyze the heterogeneity of core promoter sequences, we utilized the parameters of individual base-pair steps collected in the DiProDB database (http://diprodb.fli-leibniz.de, accessed on 27 April 2026) [11]. From the numerous properties of the ten unique double-stranded base-pair steps available in the database, we selected six parameters most suitable for evaluating the anisotropy of nucleotide sequences regarding DNA axis bending: stacking energy, roll, roll stiffness, slide, slide stiffness, and “Mobility to bend towards the major groove”. Indeed, bending anisotropy plays a crucial role in protein–DNA interactions and is therefore of great interest in this study. It is sequence-dependent and governed by the geometry and stability of individual base-pair steps [12].

Stacking energy is a component of the enthalpy of DNA formation and defines its stabilizing forces. Stiffness against roll and slide deformations also represents energy characteristics of the double-stranded structure. The roll and slide parameters characterize the mutual orientation of adjacent base pairs within a double-stranded dinucleotide [13]. The database contains several versions of these parameters; previously [5], we verified that profiles built from different parameter versions are in qualitative agreement. For this study, we chose the parameterization by Perez et al. [14] for stacking energy, roll, and slide, and the parameterization by Goni et al. [15] for stiffness variations in response to roll and slide deformations. To evaluate the stiffness of the structure regarding bending toward the major groove (using the “Mobility to bend towards the major groove” parameter), we used the parameterization by Gartenberg and Crothers [16].

**Figure 9:**
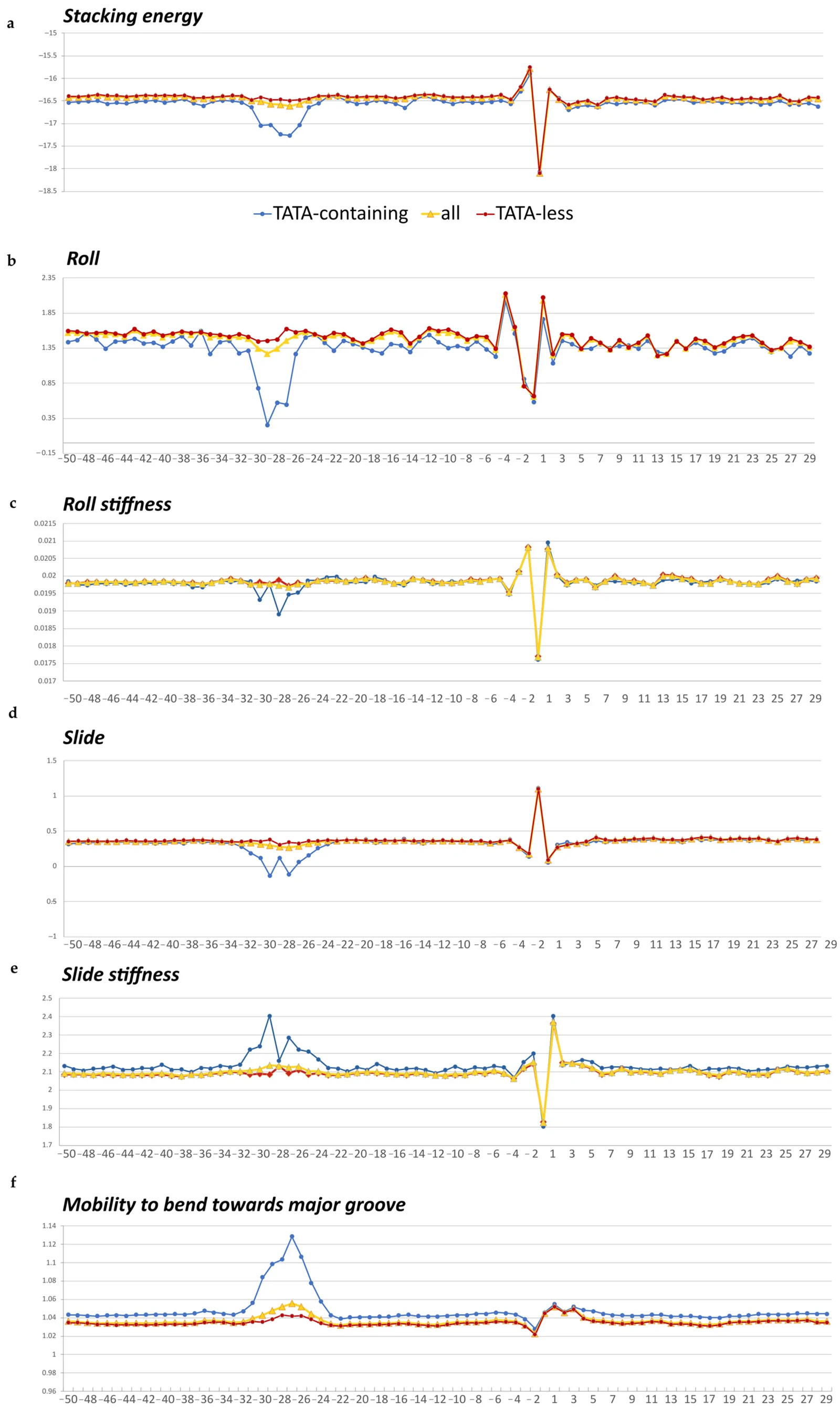
(**a**–**f**) Local variations in the values of physical and structural parameters in core promoter regions of *H. sapiens*: (**a**) Stacking energy (in kcal/mol). (**b**) Roll (in degrees). (**c**) Stiffness of the duplex structure to roll deformation (in kcal/(mol·deg)). (**d**) Slide (in Å). (**e**) Stiffness of the duplex structure to slide deformation (in kcal/(mol·Å)). (**f**) Mobility to bend towards the major groove (in mobility units).

**Figure 10:**
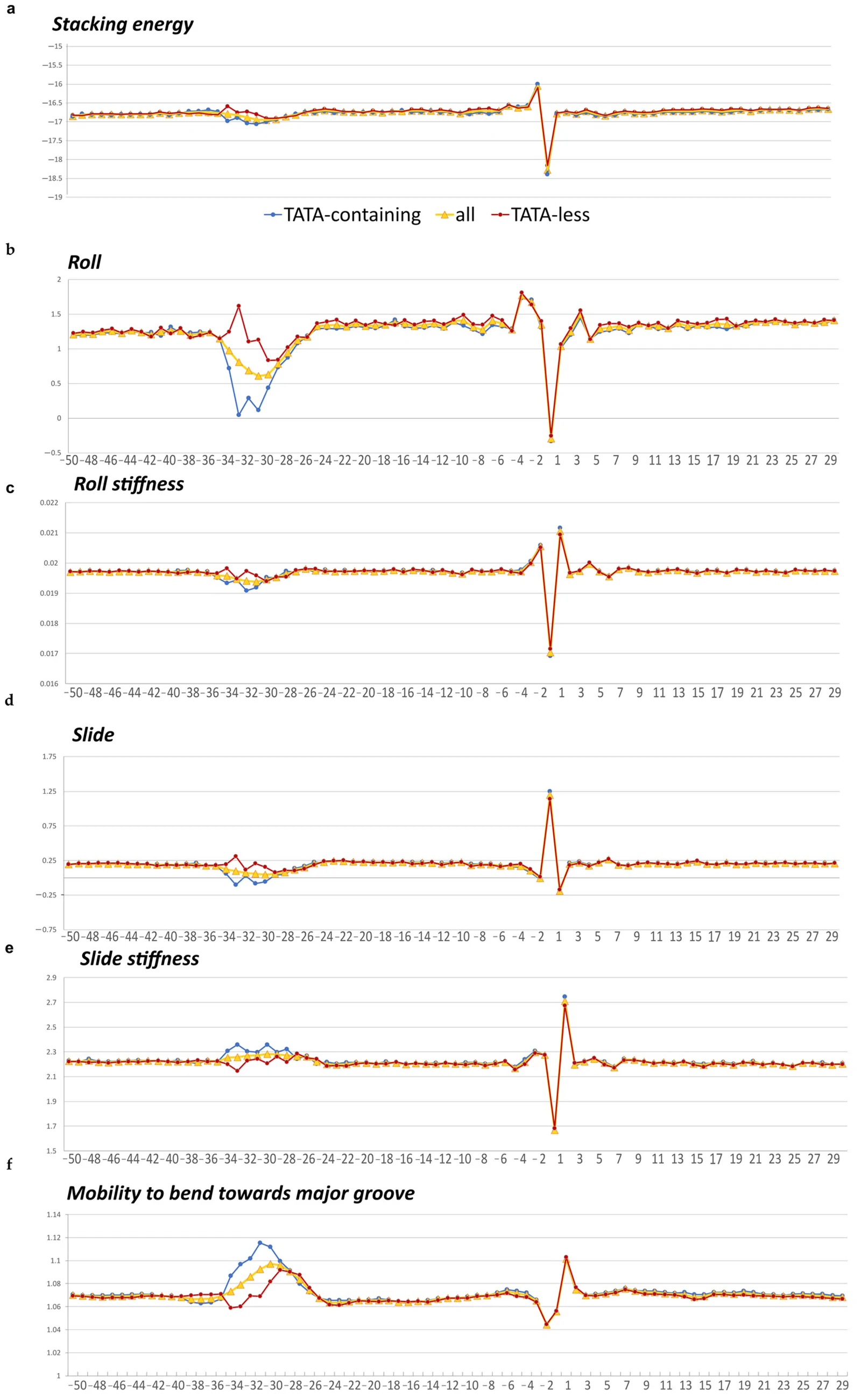
(**a**–**f**) Local variations in the values of physical and structural parameters in core promoter regions of *A. thaliana*: (**a**) Stacking energy (in kcal/mol). (**b**) Roll (in degrees). (**c**) Stiffness of the duplex structure to roll deformation (in kcal/(mol·deg)). (**d**) Slide (in Å). (**e**) Stiffness of the duplex structure to slide deformation (in kcal/(mol·Å)). (**f**) Mobility to bend towards the major groove (in mobility units).

To compare the characteristic landscapes of the complete promoter sets with their TATA-containing and TATA-less fractions, we plotted all three profiles for each parameter on a single graph. These profiles for the coding strand are shown for *H. sapiens* in Figure 9a–f and for *A. thaliana* in Figure 10a–f. Analogous data for the other organisms are provided in the Supplementary Materials (Figure S11a–f for *M. musculus*; Figure S12a–f for *C. familiaris*; Figure S13a–f for *Z. mays*; Figure S14a–f for *H. vulgare*).

These graphs show that the extrema for stacking energy, roll, and “Mobility to bend towards the major groove”—which are clearly visible in the profiles of the complete promoter sets—become markedly more pronounced in the TATA-containing promoters, but are significantly diminished or absent in the TATA-less promoters. Moreover, in TATA-containing promoters, the roll angle is low, indicating that the base pairs are nearly parallel to each other within the TATA box, whereas in TATA-less promoters, they are tilted at a much larger angle. Consequently, the octanucleotide in the TATA box of TATA-containing promoters exhibits strong stacking interactions (stacking energy of −17.3 kcal/mol in *H. sapiens*) and can easily bend toward the major groove under external influence, due to its low roll stiffness and high “Mobility to bend towards the major groove”.

In contrast, TATA-less promoters are characterized by weaker stacking (stacking energy of −16.4 kcal/mol in *H. sapiens*) and high roll angles within the TATA box. The rigidity against roll deformations also increases, while the “Mobility to bend towards the major groove” decreases, indicating that the structure of this region is less adaptable for complex formation with TBP. Thus, these profiles provide strong evidence for significant differences in the spatial structure of the octanucleotides at the TATA box position between TATA-less and TATA-containing promoters.

A nonparametric Mann–Whitney U test was used to evaluate the statistical significance of the differences in structural parameters between the TATA-containing and TATA-less promoter datasets of *H.sapiens*. The analysis was performed for six structural parameters in the TATA box region (corresponding to coordinates from −30 to −23 relative to the TSS) (see Table S1 in the Supplementary Materials).

Statistical analysis revealed that for the core promoter positions −29/−24 (and extending to −23 for most parameters), all six structural parameters exhibited highly significant differences between TATA-containing and TATA-less promoters (*p* < 0.001 for all parameters at these positions). The most pronounced effects were observed at positions −29 to −24, where the stacking energy difference reached its maximum (*p* < 0.001), indicating a more ordered structure in TATA-containing promoters that favors specific TBP binding. The roll angle at these positions was −0.6° versus 1.4° (*p* < 0.001) (see Table S1 in the Supplementary Materials). The parameter “Mobility to bend towards the major groove,” which reflects the ease of bending towards the major groove, was also significantly higher in TATA-containing promoters (1.12 vs. 1.03, *p* < 0.001), implying a lower energetic cost of DNA bending during TBP complex formation.

However, at the upstream boundary of the TATA box (position −30), the difference in stacking energy alone ceased to be statistically significant (*p* = 0.066), whereas the other five structural parameters at this same position retained their significance (*p* < 0.001). This boundary effect highlights the local nature of the structural features associated with the TATA box, with the strongest energetic stabilization confined to the internal −29 to −24 stretch rather than extending uniformly to the very edge of the octanucleotide.

Details of the statistical analysis, including bootstrap analysis of structural parameter data for TATA-containing and TATA-less promoters, are provided in the Supplementary Materials (Table S2).

However, it is important to note that the profiles in the regions between the TATA box and the TSS, as well as downstream, are highly similar between TATA-containing and TATA-less promoters and closely match the profiles of the complete sets.

Variability in conformational dynamics contributes to the heterogeneity of the double-helix structure and can be assessed using ultrasonic cleavage data [17,18,19]. These data provide information on the intensity of sugar–phosphate backbone dynamics along each of the complementary strands [20]. We used several indices [5] to describe these dynamics. Index R—the relative cleavage intensity at the central position of each of the 16 dinucleotides, index T—the relative cleavage intensity at the central position of each of the 256 tetranucleotides, and index S = (T − R)/R, which shows the influence of the neighbor nucleotides on the relative cleavage intensity of dinucleotides. If S < 0, the first and fourth nucleotides of a tetranucleotide decrease the cleavage intensity at the central step; otherwise, they increase it.

**Figure 11:**
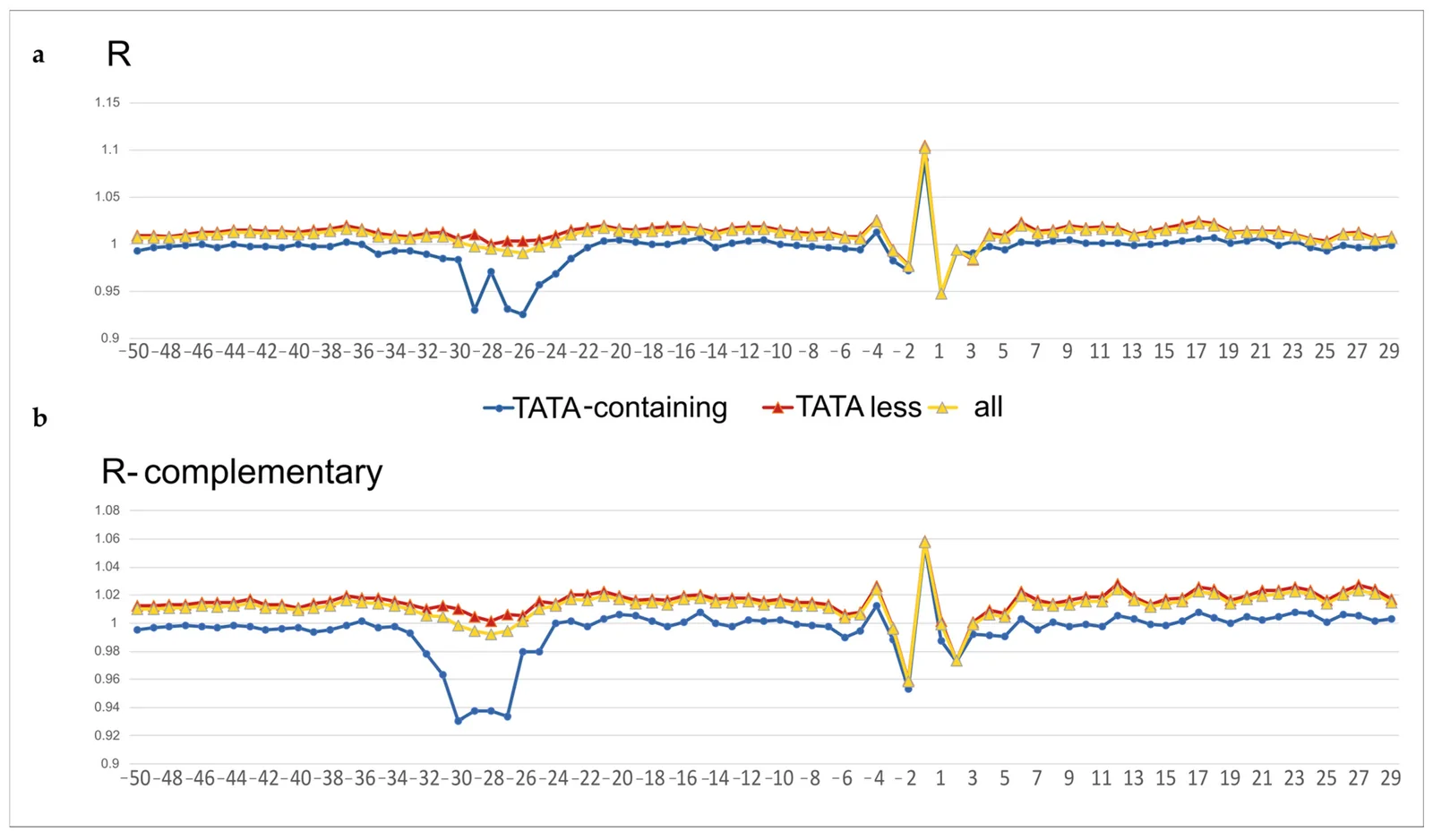
(**a**,**b**) Variation in the ultrasonic cleavage index R for both strands of *H. sapiens* core promoters. (**a**) Forward and (**b**) reverse DNA strands.

**Figure 12:**
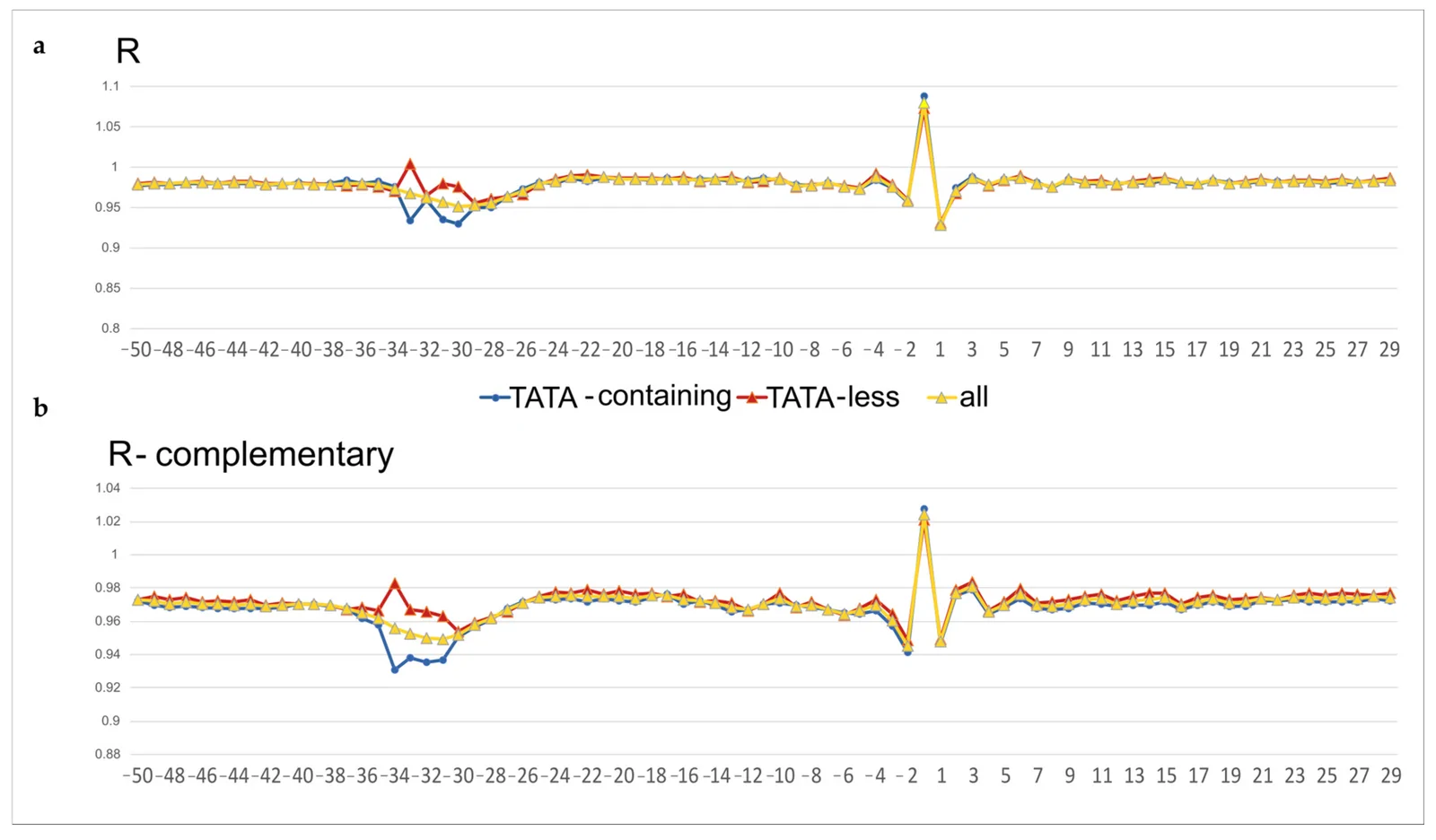
(**a**,**b**) Variation in the ultrasonic cleavage index R for both strands of *A. thaliana* core promoters. (**a**) Forward and (**b**) reverse DNA strands.

Here, we present the profiles for index R. The relative cleavage intensities of dinucleotides at different positions of the core promoters are shown for *H. sapiens* in Figure 11a,b and for *A. thaliana* in Figure 12a,b. Analogous data for the other organisms are provided in the Supplementary Materials (Figure S15a,b for *M. musculus*; Figure S16a,b for *C. familiaris*; Figure S17a,b for *Z. mays*; Figure S18a,b for *H. vulgare*).

The profiles of the relative ultrasonic cleavage intensity index R for TATA-containing promoters clearly demonstrate that conformational mobility at the TATA box position decreases sharply in all organisms without exception. Conversely, in TATA-less promoters, conformational mobility at the TATA box position increases (Figure 11a,b and Figure 12a,b and corresponding Supplementary Figures).

**Figure 13:**
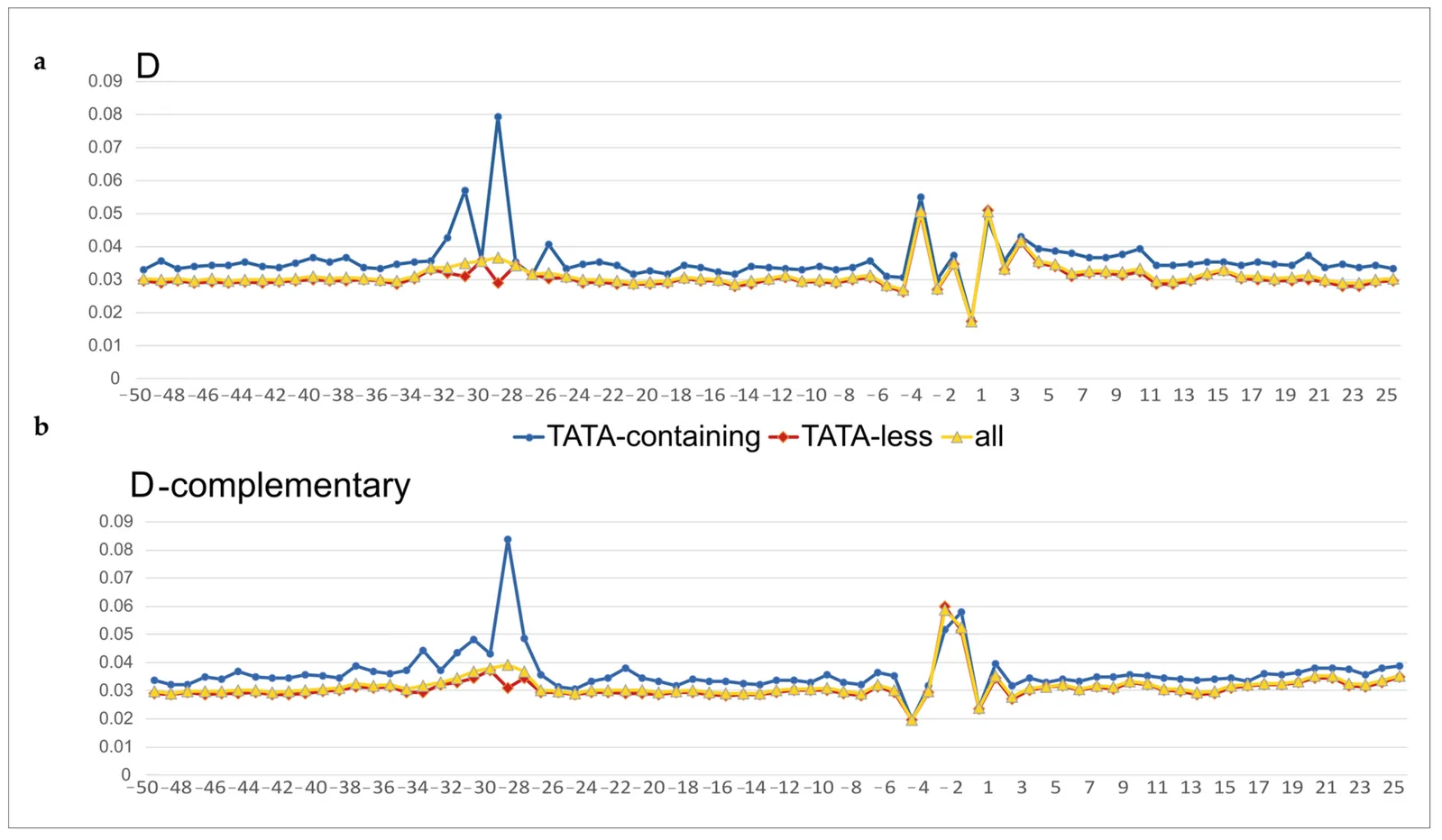
(**a**,**b**) Variation in DNase I cleavage indices in core promoters of *H. sapiens*. (**a**) Forward and (**b**) reverse DNA strands.

**Figure 14:**
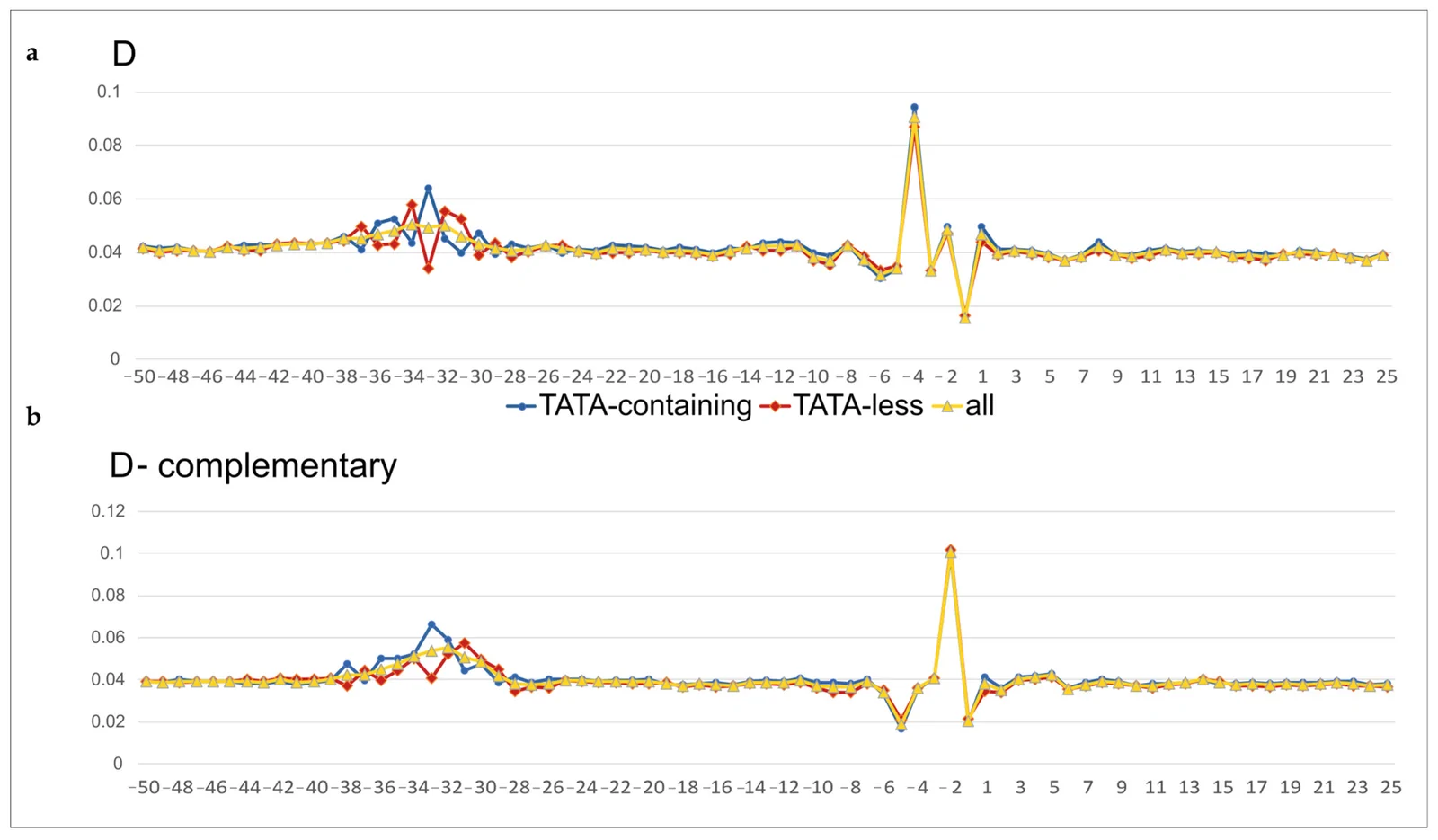
(**a**,**b**) Variation in DNase I cleavage indices in core promoters of *A. thaliana*. (**a**) Forward and (**b**) reverse DNA strands.

The profiles of DNase I cleavage for each strand of the double-stranded DNA characterize the minor-groove width [21,22,23,24]. These profiles are shown for *H. sapiens* in Figure 13a,b and for *A. thaliana* in Figure 14a,b. Analogous data for the other organisms are provided in the Supplementary Materials (Figure S19a,b for *M. musculus*; Figure S20a,b for *C. familiaris*; Figure S21a,b for *Z. mays*; Figure S22a,b for *H. vulgare*).

A marked widening of the minor groove within the TATA box of TATA-containing promoters is clearly visible in all mammals, whereas no such widening is observed in mammalian TATA-less promoters. In plants, widening of the minor groove at the TATA box position is observed in both TATA-containing and TATA-less promoters; however, in the latter, this widening is shifted downstream.

## 3. Discussion

The architecture of core promoters plays a crucial role in the assembly of the transcriptional apparatus. The course of intermolecular interactions leading to PIC formation depends on whether a complex between TBP and the TATA box can be established, as well as on the affinity of the components and the stability of the complex formed. When dividing the complete promoter sets of each organism into TATA-containing and TATA-less fractions, we based our classification on the data from [7], according to which complex formation is impossible only when the TATA box octanucleotide contains non-permissive nucleotides at specific positions (see Section 4). This approach expands the set of promoters classified as TATA-containing. Nevertheless, upon dividing the total promoter sets, we observed a significantly larger number of TATA-less promoters in all analyzed mammalian and two plant species, with the exception of *A. thaliana* (Table 1).

It is instructive to compare the proportions of TATA-containing and TATA-less promoters obtained in this study for *H. sapiens* and *M. musculus* with previous data [9], where the strict TATAWAWR consensus [25] was used as the classification criterion. According to [9], the percentage of TATA-containing promoters is 2.9% in *M. musculus* (out of 16,955 analyzed promoters) and 3.1% in *H. sapiens* (out of 29,456 promoters). In contrast, the proportions of TATA-containing promoters in our study were 15.5% for *M. musculus* (out of 25,111 promoters) and 18.05% for *H. sapiens* (out of 29,598 promoters). Thus, classifying promoters based strictly on the TATAWAWR consensus leads to a drastic reduction—nearly an order of magnitude—in the number of promoters potentially capable of forming a complex with TBP.

When separating plant promoters into two fractions, we observed that the ratio of TATA-containing to TATA-less promoters depends on whether the plant is a monocot or a dicot. In the monocots *Z. mays* and *H. vulgare*, we found 28.0% and 36.45% TATA-containing promoters, respectively, whereas in the dicot *A. thaliana*, this proportion was 51.30%. A similar percentage of TATA-containing promoters in *A. thaliana* (57%) was reported previously [26]. This is significantly higher than the estimate obtained later by [27], where TATA-containing promoters in *A. thaliana* were defined by the presence of the TATATATA consensus identified using MEME. Comparing these data indicates that the strict TATAWAWR consensus criterion should not be used to estimate the proportions of TATA-containing and TATA-less promoters in plants either.

Primary structure analysis revealed that TATA-containing mammalian promoters have a low GC content, whereas a high GC content is a distinctive feature of TATA-less mammalian promoters. These findings are clearly visible both upstream and downstream of the TATA box (Figure 3, Figure 4, Figure 5, Figure 6, Figure 7 and Figure 8). The inversion in the frequency of G and C nucleotides at −40 bp in the profiles of TATA-less mammalian promoters can be attributed to the presence of the DREu motif. A similar inversion at −18 bp, observed in *H. sapiens* and *M. musculus* but absent in *C. familiaris*, can be explained by the DREd motif.

In contrast, in plant TATA-less promoters, the increase in GC content is very small in the monocots *Z. mays* and *H. vulgare* (Figure 7 and Figure 8) and is completely absent in the dicot *A. thaliana* (Figure 6). Thus, in terms of GC content, TATA-less promoters of mammals differ significantly from those of plants.

However, the architectural differences between TATA-containing and TATA-less promoters are very similar in both plants and mammals, as evidenced by their profiles of spatial structure and dynamics. The distributions of all six structural parameters in the TATA box of TATA-containing and TATA-less promoters differ significantly (see the Supplementary Materials).

Based on the structural and dynamic parameter profiles, one can unambiguously classify a promoter set as either TATA-containing or TATA-less. The energy characteristics—stacking energy, roll stiffness, slide stiffness, and the “Mobility to bend towards the major groove”—differ in the depth of their potential wells, while the structural parameters, roll and slide, vary in the direction of their extrema. The “Mobility to bend towards the major groove” parameter is particularly important as it provides an estimate of the local bending stiffness of the double helix. The formation of the TBP-TATA box complex in TATA-containing promoters of both mammals and plants is facilitated by the ability of the TATA box octanucleotide to bend toward the major groove with low energy costs while maintaining an ordered structure with strong stacking interactions.

This is consistent with well known results of X-ray crystallographic analysis of TBP complexes with octanucleotides with TATAWAWR consensus, which made it possible to determine the main features of DNA in complexes—the degree of its deformation and the widening of the minor groove along which contacts with the TBP molecule occur [28,29,30].

It is known that DNA–protein interactions are generally less sequence-selective in the minor groove than in the major groove [31]. Indeed, TBP exhibits less than a 10^3^-fold preference for binding the TATAAAAG sequence compared to nonspecific yeast genomic DNA. It has been previously shown that the binding efficiency of the octanucleotide to TBP is significantly influenced by its bending rigidity toward the major groove [32,33]. This suggests that the energetic cost of bending the octanucleotide toward the major groove may influence transcription efficiency. In this work, we went beyond the conventional notion of the exclusivity of the TATAWAWR consensus defining the TATA-containing promoter. This approach was inspired by the results of [7], in which X-ray structural analysis was accompanied by an assessment of promoter efficiency. However, the complexation of a large number of octanucleotides with TBP, which, according to [7], we attribute to forming TATA-containing promoters, has not been experimentally studied. Therefore, the possibility of PIC formation involving these octanucleotides has not been experimentally confirmed. However, the structural, sonication, and DNase I binding profiles of these octanucleotides suggest that the presence of all these octanucleotides located in the TATA box position may define the promoter as TATA-containing.

It can be assumed that the differences in the affinity of octanucleotides for TBP can significantly influence the formation of the PIC, from the classical pathway (driven by the TATAWAWR consensus) to unstable variants of formation and decay, which can significantly change the kinetics of PIC formation.

In [34], the in vitro system for yeast demonstrates the importance of the TATA-like sequence for many TATA-less promoters. Moreover, it was shown that the presence or absence of the TATA element is likely not the only feature that distinguishes TATA-containing and TATA-less promoters.

The mechanism of PIC formation in TATA-less promoters must be fundamentally different from that in TATA-containing promoters, as TBP cannot form a complex within the TATA box region. Sterically inappropriate contacts prevent this complexation. In mammals, a motif around −40 bp appears to act as an anchor for TFIID complex formation with the core promoter. In contrast, we found no evidence of such anchoring motifs upstream of the TATA box in plants’ core promoters. Therefore, it may be assumed that the mechanism of mRNA transcription driven by TATA-less promoters likely differs between mammals and plants.

## 4. Materials and Methods

We analyzed promoter sets from six organisms: mammals (*H. sapiens*, 29,598 promoters; *M. musculus*, 25,111 promoters; *C. familiaris*, 7545 promoters) and plants (*A. thaliana*, 22,703 promoters; *Z. mays*, 17,081 promoters; *H. vulgare*, 21,193 promoters). These were retrieved from the EPDnew section of the Eukaryotic Promoter Database (EPD; http://epd.vital-it.ch, accessed on 20 April 2026). We verified that all sequences were exactly 80 nucleotides in length with strictly defined boundaries.

### 4.1. Splitting Promoters into Datasets

#### 4.1.1. Splitting Promoters into TATA-Containing and TATA-Less Datasets

According to X-ray structural data [7], complex formation between an octanucleotide and the TATA-binding protein (TBP) is possible only if the permitted nucleotides are present at all eight positions. At positions 2, 4, and 5, cytosine (C) and guanine (G) are not permitted. At position 3, only G is not permitted. Therefore, a promoter is classified as TATA-containing if it meets the following criteria: any nucleotide (A, T, G, or C) at positions 1, 6, 7, and 8; only A or T at positions 2, 4, and 5; and A, T, or C at position 3. Otherwise, the promoter is classified as TATA-less.

Therefore, a promoter was classified as TATA-containing if the octanucleotide in the TATA box position matched the following regular expression: [ATGC][AT][ATC][AT][AT][ATGC][ATGC][ATGC]. Promoters that did not match this pattern were assigned to the TATA-less fraction. This rule was applied to each unique promoter sequence using a custom Python 3.10 script.

#### 4.1.2. Splitting Promoters into TATA-Consensus and TATA-Non-Consensus Datasets

We compared the octanucleotides in the TATA box position with the TATAWAWR consensus. If the octanucleotide corresponded to the TATAWAWR consensus exactly, we called it a promoter with the TATA consensus. Otherwise, it was called a TATA-non-consensus promoter.

### 4.2. Profile Construction

The X-axis of each profile represents the position relative to the TSS, which is denoted as +1 bp, with negative and positive numbers indicating the upstream and downstream regions, respectively. The Y-axis represents the mean value of a selected characteristic calculated from the corresponding datasets.

For sequence characteristics defined at the mononucleotide level, the counts of each nucleotide (A, C, G, T) across all core promoters of a given species were summed for each of the 80 positions on the X-axis (−50, −49, …, −1, +1, +2, …, +30), and the resulting sum was divided by the total number of promoters.

For physical or structural characteristics defined at the base-pair step level, or for ultrasonic cleavage rates at the dinucleotide level, the values were summed across the 79 positions on the X-axis (−49, −48, …, −1, +1, +2, …, +30). These values correspond to dinucleotides at positions [(−50, −49), (−49, −48), …, (−1, +1), …, (+29, +30)] and were obtained from DiProDB (for physical and structural characteristics) or from the Supporting Information of [17] (for dinucleotide-level ultrasonic cleavage rates). The resulting sum was then divided by the total number of promoters.

For DNase cleavage rates at the hexanucleotide level, the values were summed across the 75 positions on the X-axis (−47, −46, …, −1, +1, +2, …, +27). These values correspond to hexanucleotides at positions [(−50, −49, −48, −47, −46, −45), …, (−3, −2, −1, +1, +2, +3), …, (+25, +26, +27, +28, +29, +30)] and were obtained from the Supporting Information of [20]. The resulting sum was divided by the total number of promoters.

To analyze the structural and mechanical properties of the core promoter sequences, we used numerical parameterization indices for the ten unique double-stranded base-pair steps, which were retrieved from the DiProDB database (http://diprodb.fli-leibniz.de, accessed on 26 April 2026) [11]. To construct profiles for variations in stacking energy and the base-pair step parameters roll and slide, we used the parameterization by Perez et al. [14]. For profiles of DNA double-helix stiffness variations in response to roll and slide deformations, we used the parameterization by Goni et al. [15]. To construct profiles of the “Mobility to bend towards the major groove” parameter, we used the parameterization by Gartenberg and Crothers [16].

Profile construction was performed using custom scripts written in Python 3.10. Sequence logos were generated using the WebLogo tool (http://weblogo.threeplusone.com, accessed on 28 April 2026).

### 4.3. Statistical Analysis

All statistical analyses were performed using Python (version 3.10) with the NumPy (version 1.24) and SciPy (version 1.11) libraries. To compare the distributions of structural parameters between TATA-containing and TATA-less promoters, we used the nonparametric Mann–Whitney U test (two-sided) to compare the median values of each structural parameter between the two groups at each dinucleotide position. This test was chosen because the data did not follow a normal distribution (Shapiro–Wilk test, *p* < 0.05 for most parameters). A *p*-value < 0.05 was considered statistically significant.

## 5. Conclusions

The aim of this study was to identify differences in the primary and spatial structure of naked DNA in TATA-containing and TATA-less promoters of Pol II. We analyzed the −50 to +30 bp fragment of core promoters, which includes two key regions: the initiator (Inr) hexanucleotide around the TSS and the TATA box, as well as a variety of motifs, whose specific sets vary depending on the promoter type.

Promoters from three mammalian (*H. sapiens*, *M. musculus*, and *C. familiaris*) and three plant (*A. thaliana*, *Z. mays*, and *H. vulgare*) organisms were analyzed. For this purpose, we retrieved promoter sets from the EPDnew section of the Eukaryotic Promoter Database (EPD; http://epd.vital-it.ch, accessed on 20 April 2026) in which the promoters of each organism were aligned relative to the TSS. We separated the complete sets of promoters from each organism based on the ability of the octanucleotide representing the TATA box to form a complex with TBP. To identify promoters potentially capable of forming a complex with TBP, we applied a structure-based definition derived from high-resolution co-crystal structures of TBP with 10 natural TATA-element variants [7]. A promoter was classified as TATA-containing if the octanucleotide in the TATA box position matched the following regular expression: [ATGC][AT][ATC][AT][AT][ATGC][ATGC][ATGC]. Promoters that did not match this pattern were assigned to the TATA-less fraction. This rule was applied to each unique promoter sequence using a custom Python 3.10 script. Thus, we obtained larger samples of TATA-containing promoters than when splitting according to the TATAWAWR consensus criterion. However, it turned out that the sample sizes for TATA-containing core promoters were significantly smaller than those for TATA-less ones.

The comparison of the primary structure of promoters was carried out on the basis of mononucleotide occurrence frequencies (in percentages) at each position along the coding strand, as well as using sequence logo representations. As a result, differences in the primary structure between mammals and plants were revealed. Namely, it was shown that in mammals there is no predominance of TA in the TATA box of TATA-less promoters, whereas in plants TATA-less promoters preserve a slight predominance of TA in the TATA box. Also the GC content around the TATA box is notably higher in TATA-less promoters than in TATA-containing ones in all mammals, but not in plants.

We performed spatial structure analysis based on a comparison of the profiles of the structural parameters of dinucleotides (stacking energy, roll, stiffness of the duplex structure to roll deformation, slide, stiffness of the duplex structure to slide deformation and mobility to bend towards the major groove), as well as on ultrasonic cleavage profiles and DNase I cleavage profiles.

As a result, it was found that the spatial structure and dynamics—and hence the architecture of promoters—of TATA-containing and TATA-less core promoters differ in both mammals and plants. The octanucleotide at the TATA box position in TATA-less promoters exhibits weaker stacking interactions, high roll angles, increased conformational dynamics, and a significantly less pronounced reduction in bending rigidity toward the major groove compared to those in TATA-containing promoters. The differences between the structural parameters of dinucleotides in TATA-containing and TATA-less promoters are statistically significant.

## Figures and Tables

**Table 1:** Proportions of TATA-containing and TATA-less promoter fractions classified according to the sequence restriction rules described in [7].

Organism	*H. sapiens*	*M. musculus*	*C. familiaris*	*A. thaliana*	*Z. mays*	*H. vulgare*
TATA-containing	18.05%	15.50%	14.27%	51.30%	28.00%	36.45%
TATA-less	81.95%	84.50%	85.73%	48.70%	72.00%	63.55%

**Table 2:** Proportions of TATA-consensus and TATA-non-consensus promoters classified based on the presence of the TATAWAWR consensus in the TATA box positions.

Organism	*H. sapiens*	*M. musculus*	*C. familiaris*	*A. thaliana*	*Z. mays*	*H. vulgare*
TATA-Consensus	0.59%	0.92%	0.84%	3.89%	1.69%	2.23%
TATA-non- Consensus	99.41%	99.08%	99.16%	96.11%	98.31%	97.77%

## Data Availability

These data were derived from the following resources available in the public domain: http://epd.vital-it.ch, http://diprodb.fli-leibniz.de.

## Supplementary Materials

The following supporting information can be downloaded at https://www.mdpi.com/article/10.3390/ijms27177735/s1.
