# Supplementary material for: Structural Features of DNA in TATA-Containing and TATA-Less Core Promoters of RNA Polymerase II Differ
Source: Int J Mol Sci. 2026 Aug 28;27(17):7735. doi: 10.3390/ijms27177735 (PMC13566119; doi:10.3390/ijms27177735)
Supplement: Supplementary file 1 [file ijms-27-07735-s001.zip › ijms-4482997-supplementary.pdf]

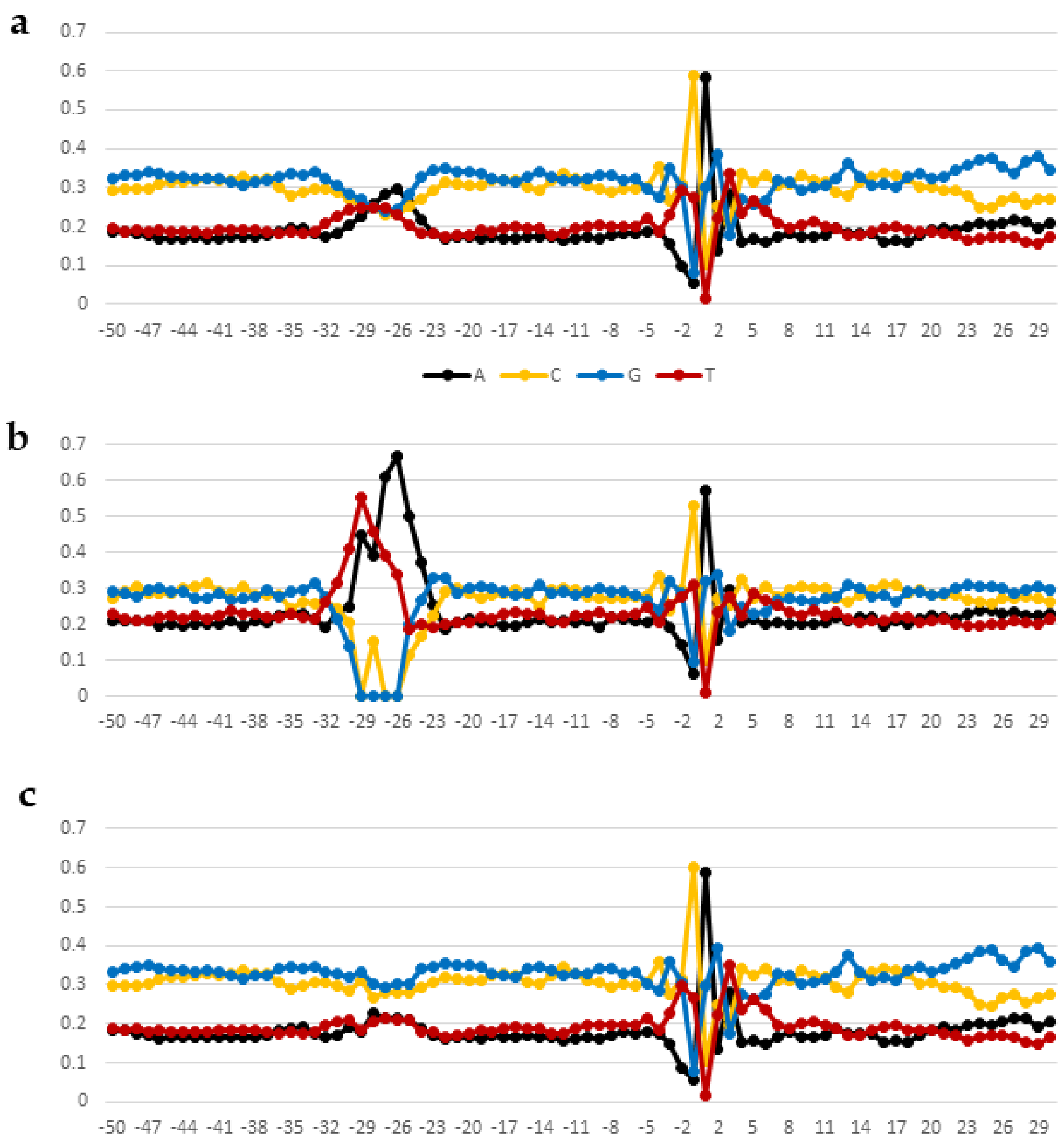


**Figure S1 (a-c).** Mononucleotide occurrence frequencies (in percentages) at each position along the coding strand of *M. musculus*: (a) complete set of promoters; (b) TATA-containing promoters; (c) TATA-less promoters.

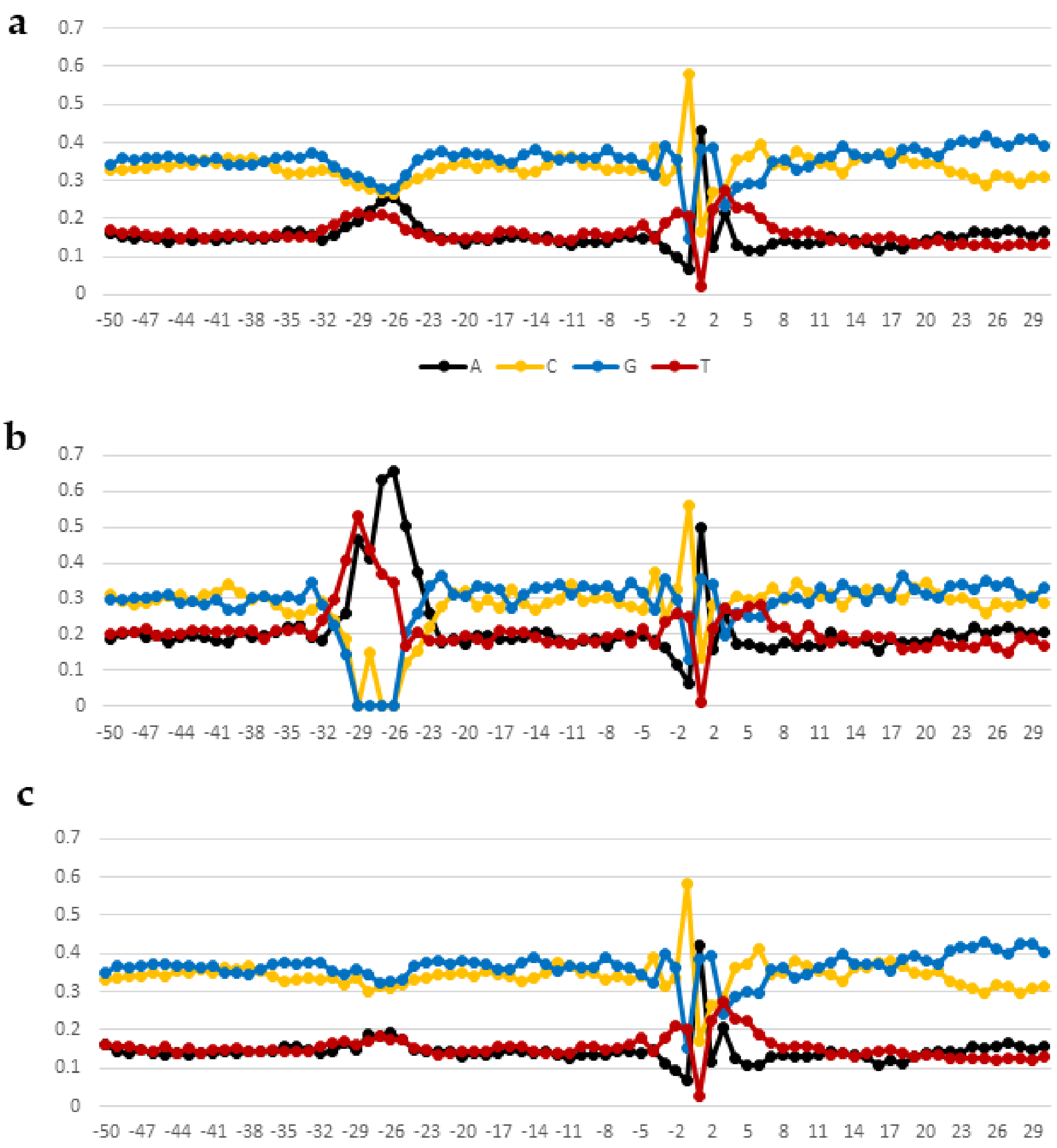


**Figure S2 (a-c).** Mononucleotide occurrence frequencies (in percentages) at each position along the coding strand of *C. familiaris*: (a) complete set of promoters; (b) TATA-containing promoters; (c) TATA-less promoters.

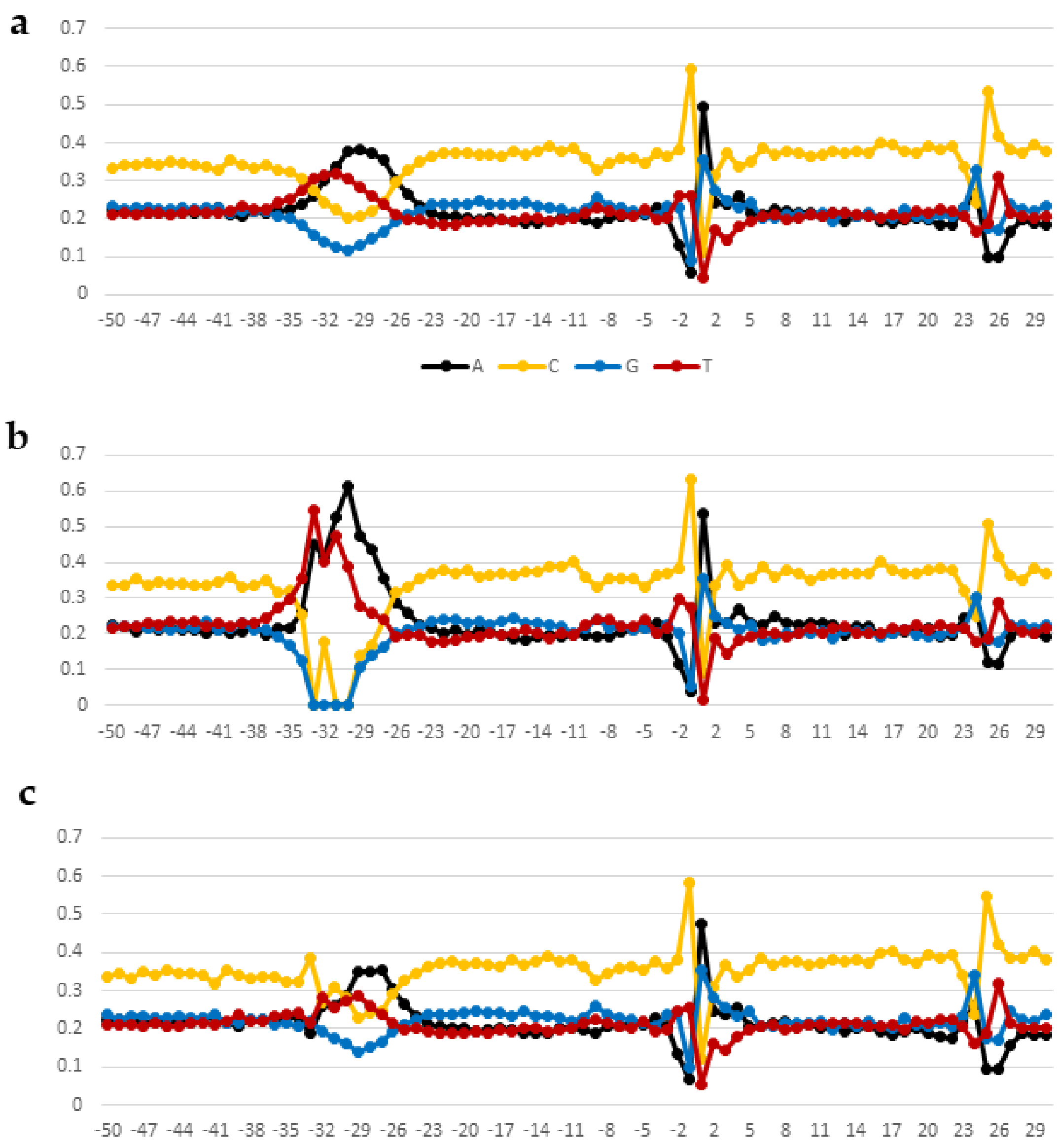


**Figure S3 (a-c).** Mononucleotide occurrence frequencies (in percentages) at each position along the coding strand of *Z. mays*: (a) complete set of promoters; (b) TATA-containing promoters; (c) TATA-less promoters.

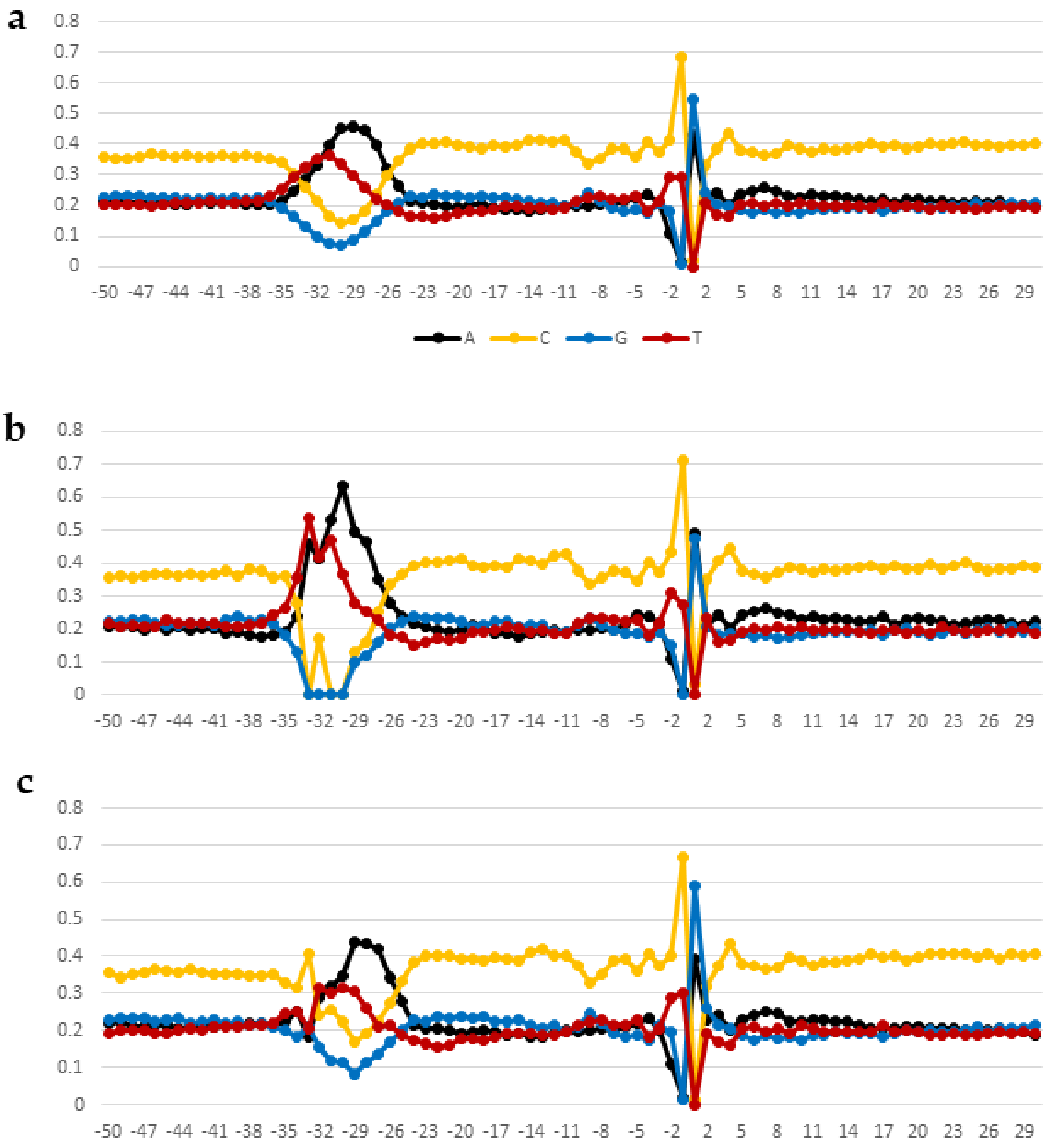


**Figure S4 (a-c).** Mononucleotide occurrence frequencies (in percentages) at each position along the coding strand of *H. vulgare*: (a) complete set of promoters; (b) TATA-containing promoters; (c) TATA-less promoters.

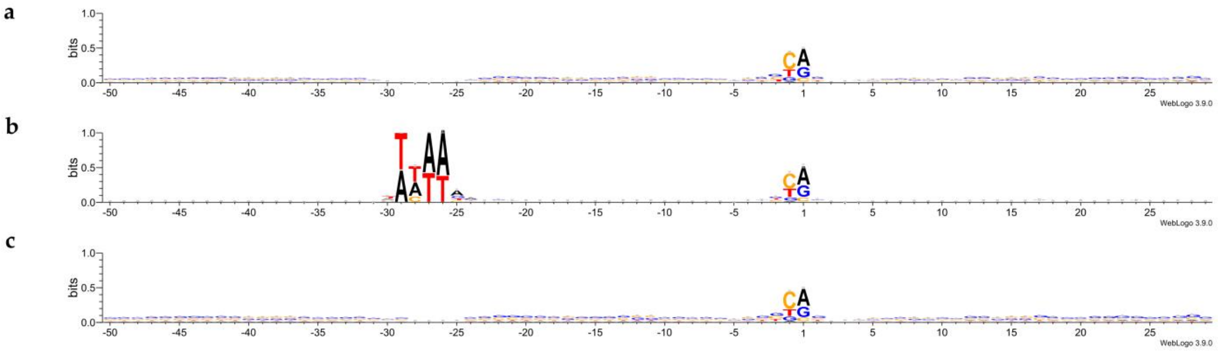


**Figure S5 (a, b).**LOGO representation with informational content 1.0 for all (a), TATA-containing (b) and TATA-less (c) promoters of *H. sapiens*.

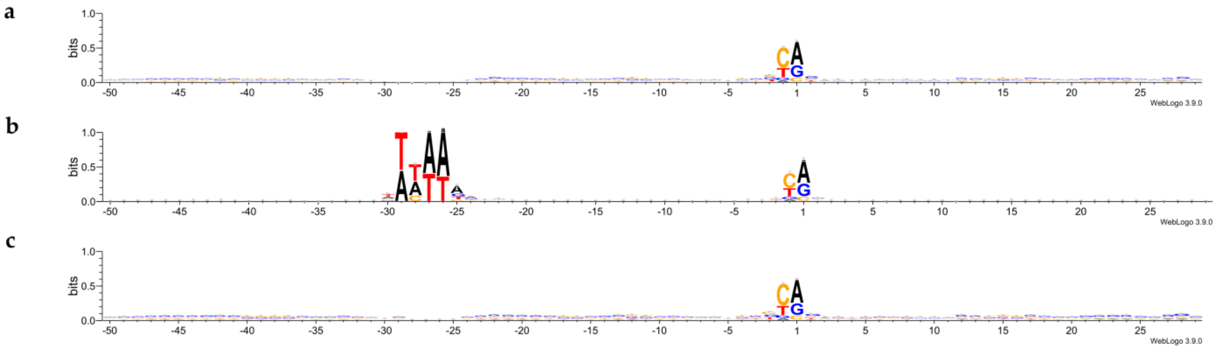


**Figure S6 (a, b).**LOGO representation with informational content 1.0 for all (a), TATA-containing (b) and TATA-less (c)promoters of *M. musculus*.

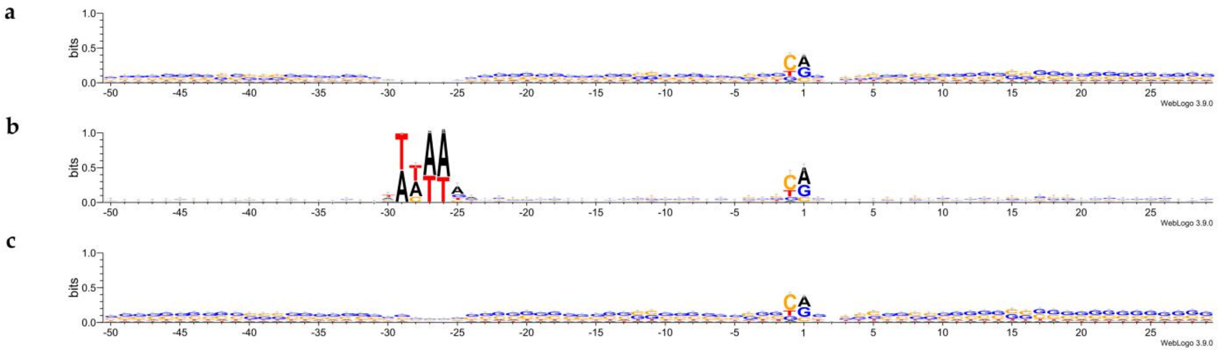


**Figure S7 (a, b).**LOGO representation with informational content 1.0 for all (a), TATA-containing (b) and TATA-less (c)promoters of *C. familiaris*.

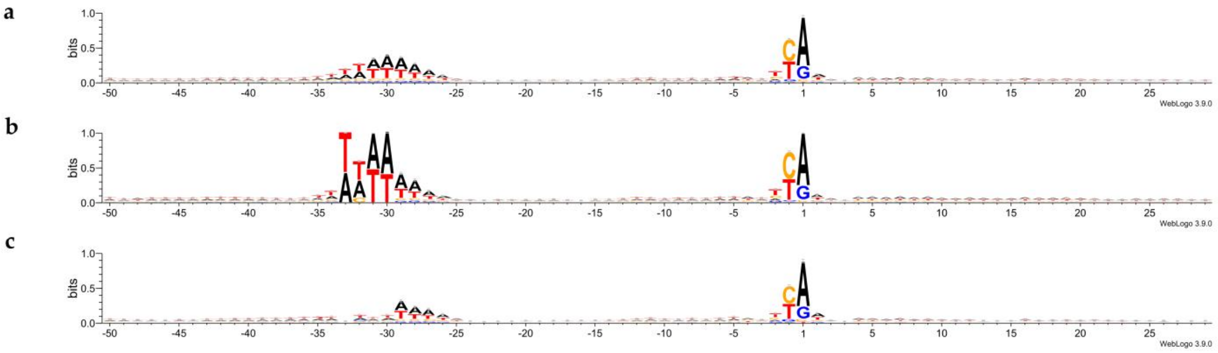

**Figure S8 (a, b).**LOGO representation with informational content 1.0 for all (a), TATA-containing (b) and TATA-less (c)promoters of *A. thaliana*.

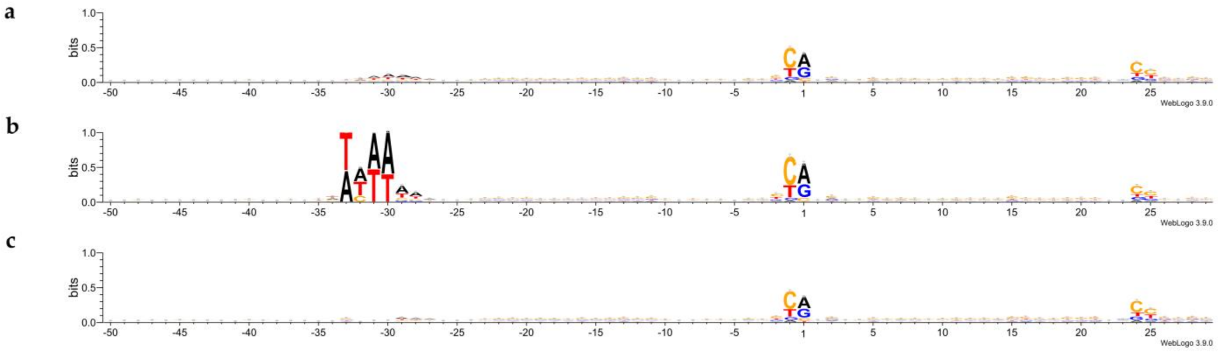

**Figure S9 (a, b).**LOGO representation with informational content 1.0 for all (a), TATA-containing (b) and TATA-less (c)promoters of *Z. mays*.

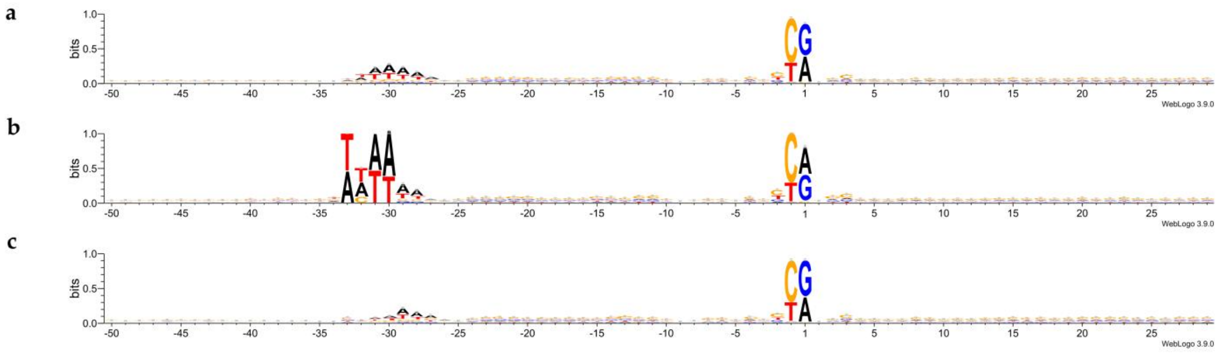

**Figure S10(a, b).**LOGO representation with informational content 1.0 for all (a), TATA-containing (b) and TATA-less (c)promoters of *H. vulgare*.

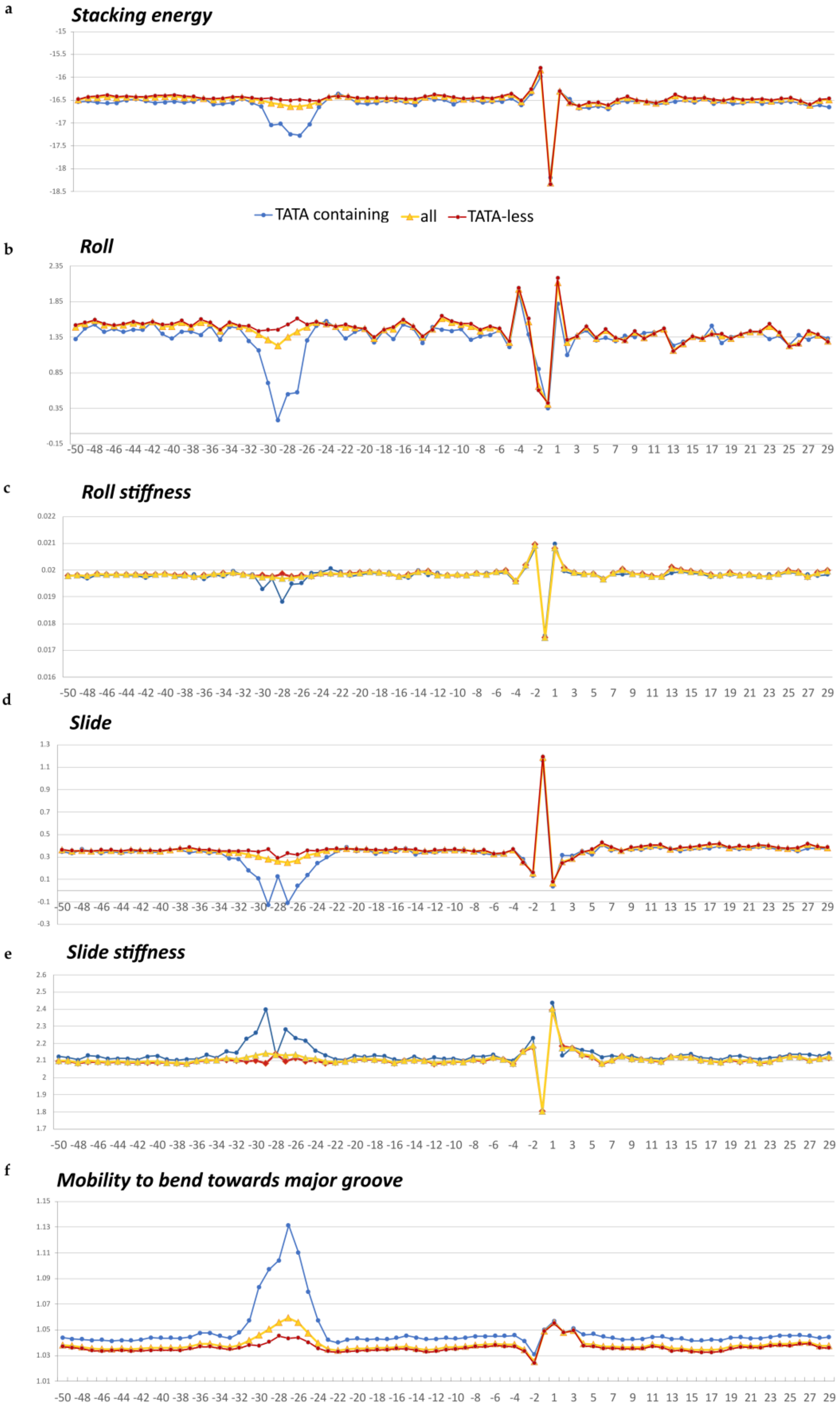


**Figure S11 (a-f).** Local variations of the values of physical and structural parameters in core promoter regions of *M. musculus*. (a) Stacking energy (in kcal/mol). (b) Roll (in degrees). (c) Stiffness of the duplex structure to Roll deformation (in kcal/(mol·deg)). (d) Slide (in Å). (e) Stiffness of the duplex structure to Slide deformation (in kcal/(mol·Å)). (f) Mobility to bend towards the major groove (in mobility units).

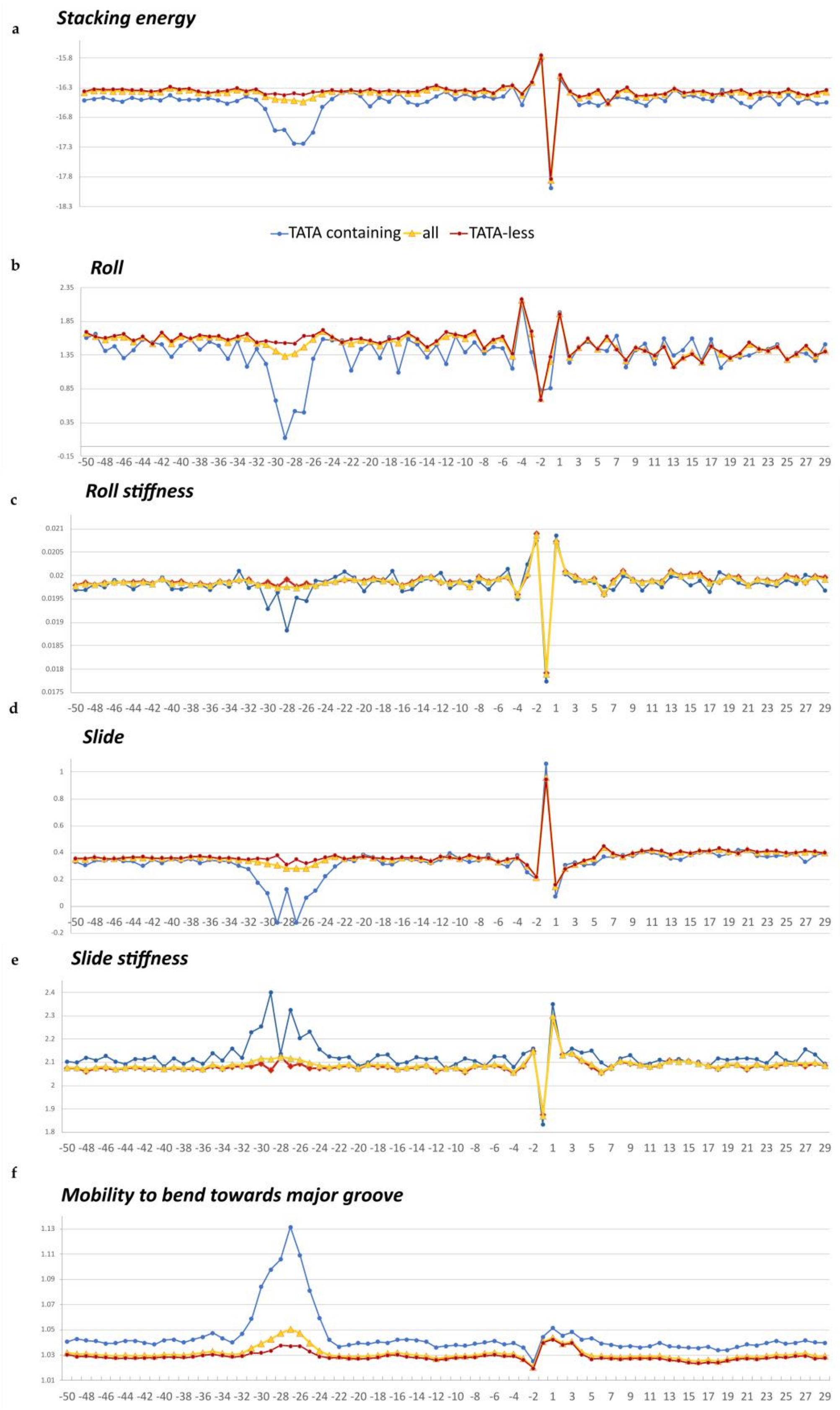


**Figure S12(a-f).**Local variations of the values of physical and structural parameters in core promoter regions of *C. familiaris*. (a) Stacking energy (in kcal/mol). (b) Roll (in degrees). (c) Stiffness of the duplex structure to Roll deformation (in kcal/(mol·deg)). (d) Slide (in Å). (e) Stiffness of the duplex structure to Slide deformation (in kcal/(mol·Å)). (f) Mobility to bend towards the major groove (in mobility units).

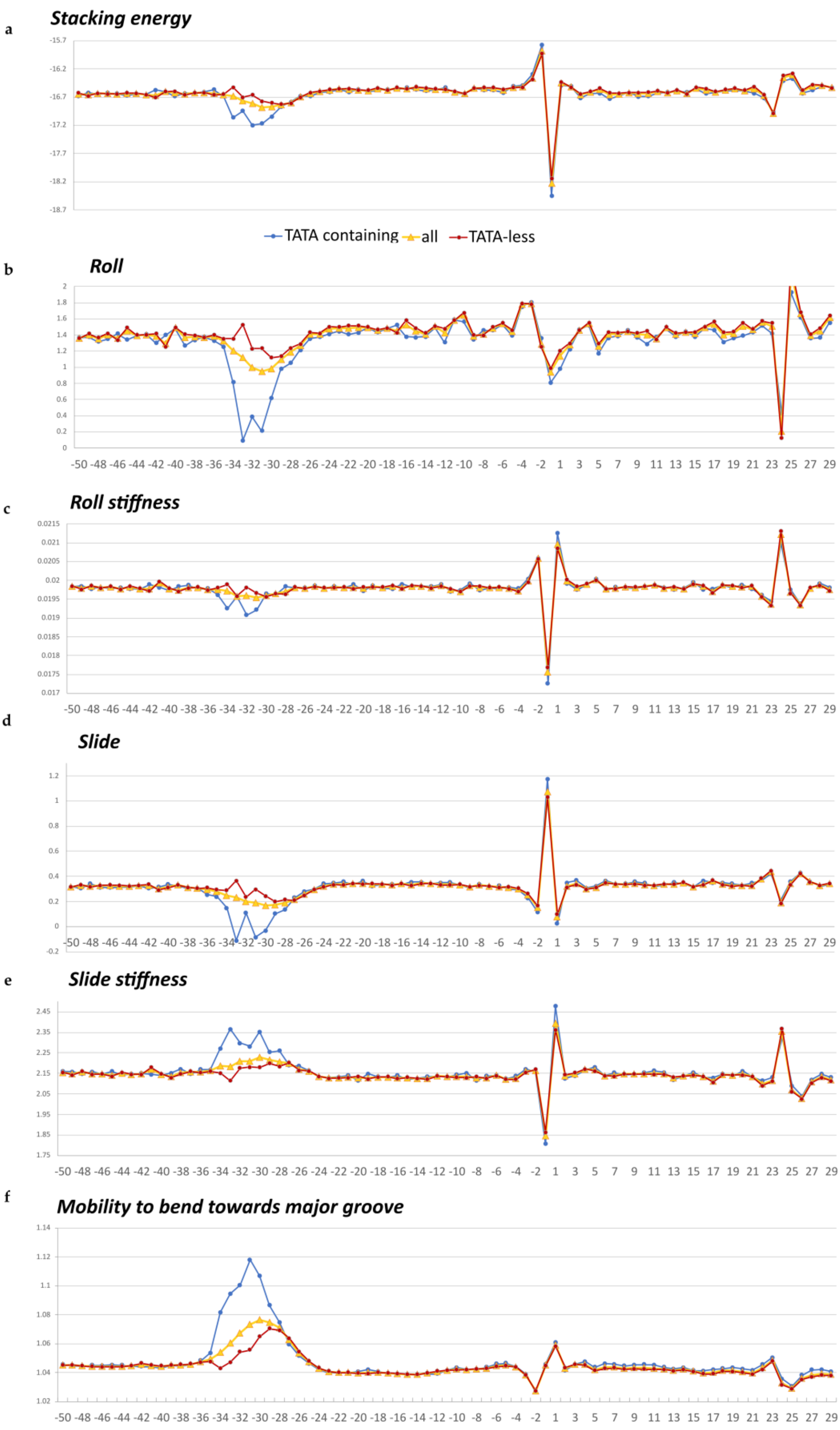


**Figure S13(a-f).**Local variations of the values of physical and structural parameters in core promoter regions of *Z. mays*. (a) Stacking energy (in kcal/mol). (b) Roll (in degrees). (c) Stiffness of the duplex structure to Roll deformation (in kcal/(mol·deg)). (d) Slide (in Å). (e) Stiffness of the duplex structure to Slide deformation (in kcal/(mol·Å)). (f) Mobility to bend towards the major groove (in mobility units).

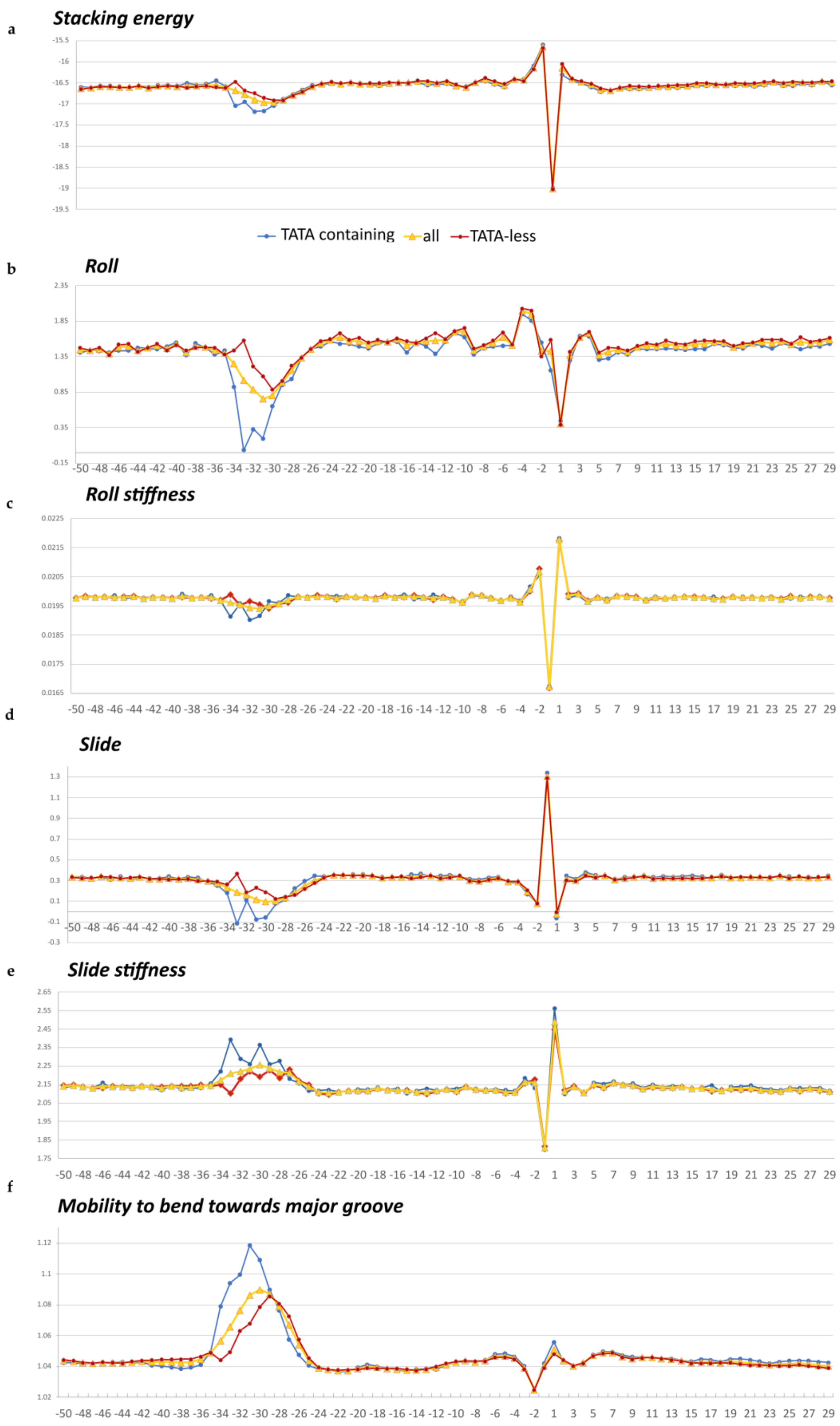


**Figure S14(a-f).**Local variations of the values of physical and structural parameters in core promoter regions of *H. vulgare*. (a) Stacking energy (in kcal/mol). (b) Roll (in degrees). (c) Stiffness of the duplex structure to Roll deformation (in kcal/(mol·deg)). (d) Slide (in Å). (e) Stiffness of the duplex structure to Slide deformation (in kcal/(mol·Å)). (f) Mobility to bend towards the major groove (in mobility units).

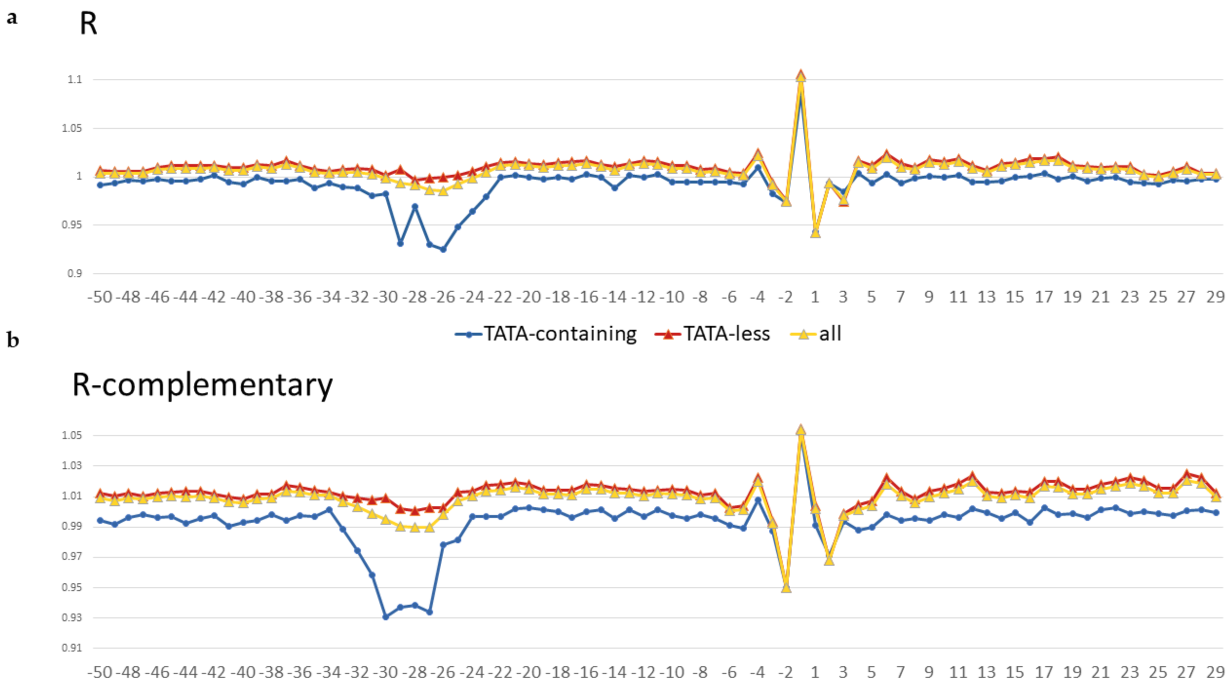


**Figure S15(a,b).** Variation of the ultrasonic cleavage index R for both strands of *M. musculus*core promoters.

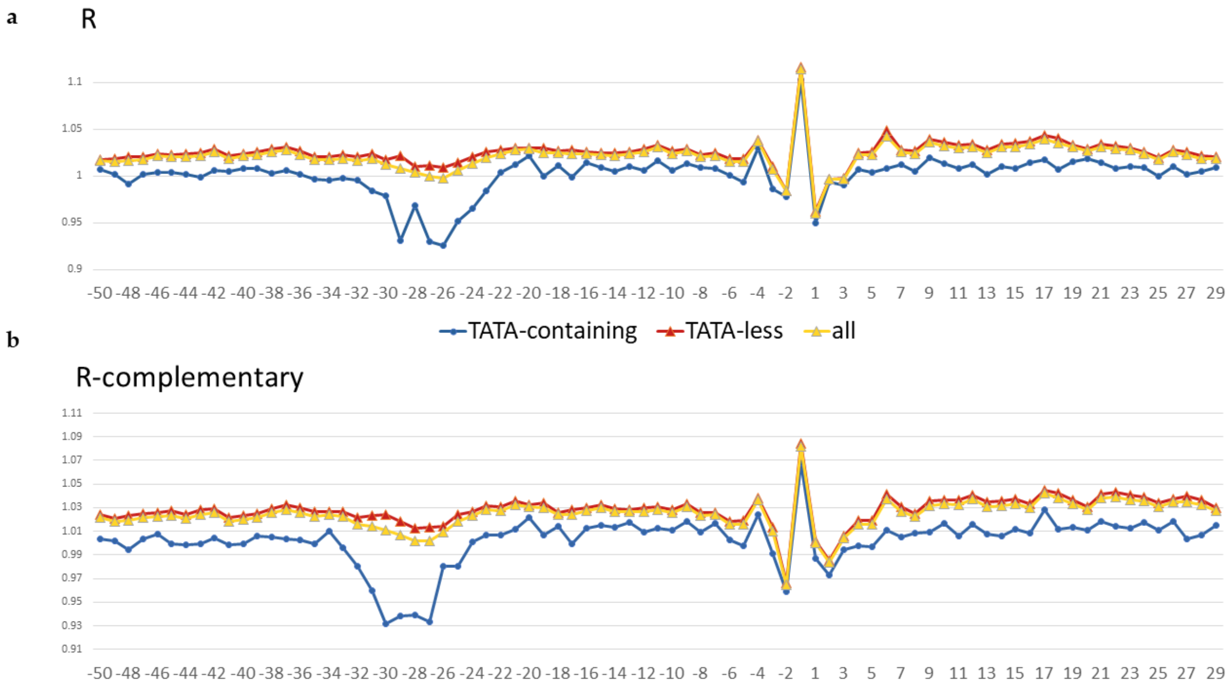


**Figure S16(a,b).** Variation of the ultrasonic cleavage index R for both strands of *C. familiaris*core promoters.

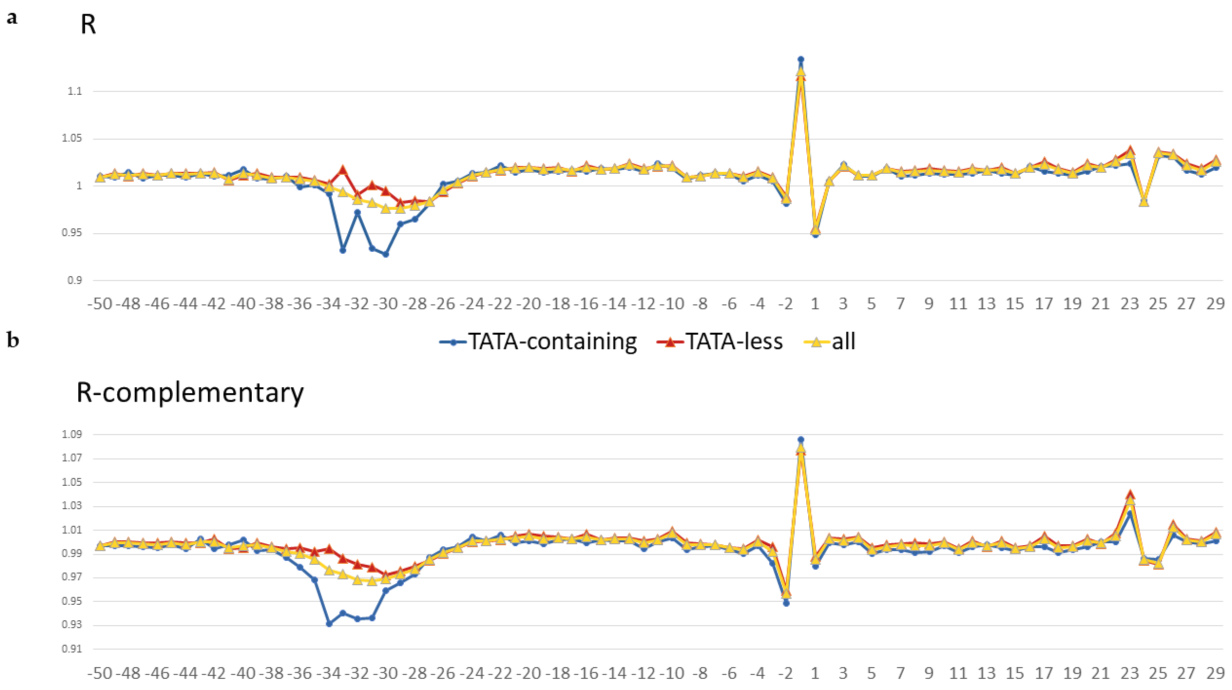


**Figure S17(a,b).** Variation of the ultrasonic cleavage index R for both strands of *Z. mays*core promoters.

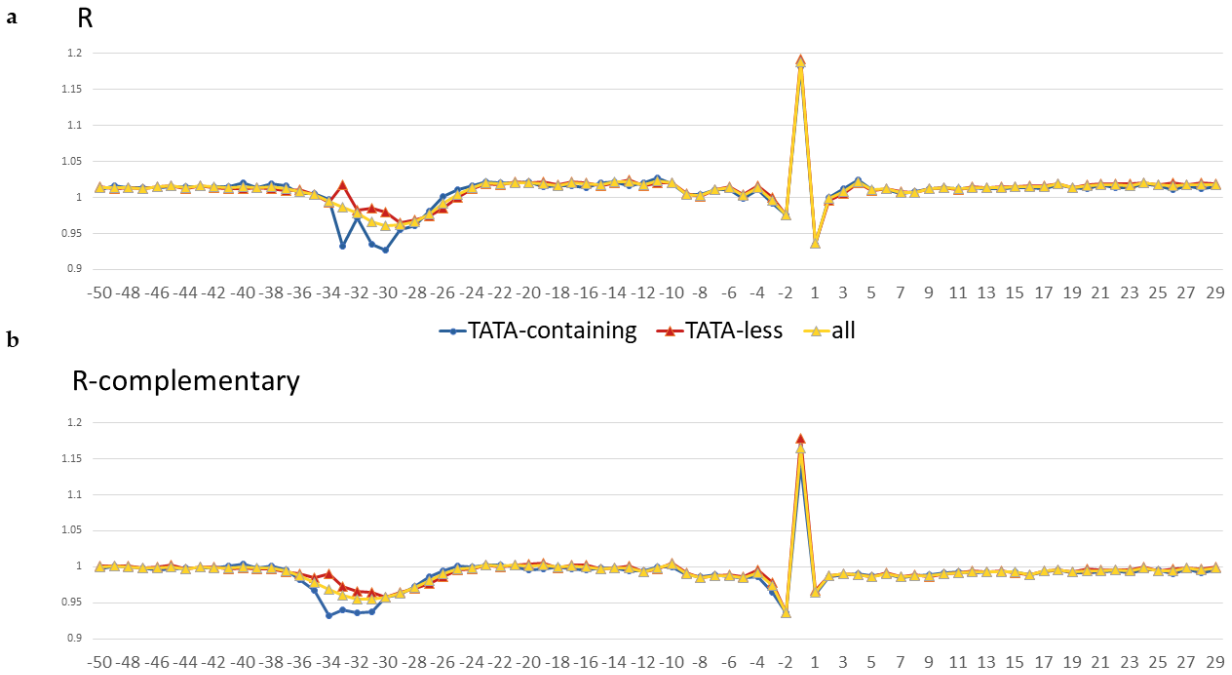


**Figure S18(a,b).** Variation of the ultrasonic cleavage index R for both strands of *H. vulgare*core promoters.

a

b

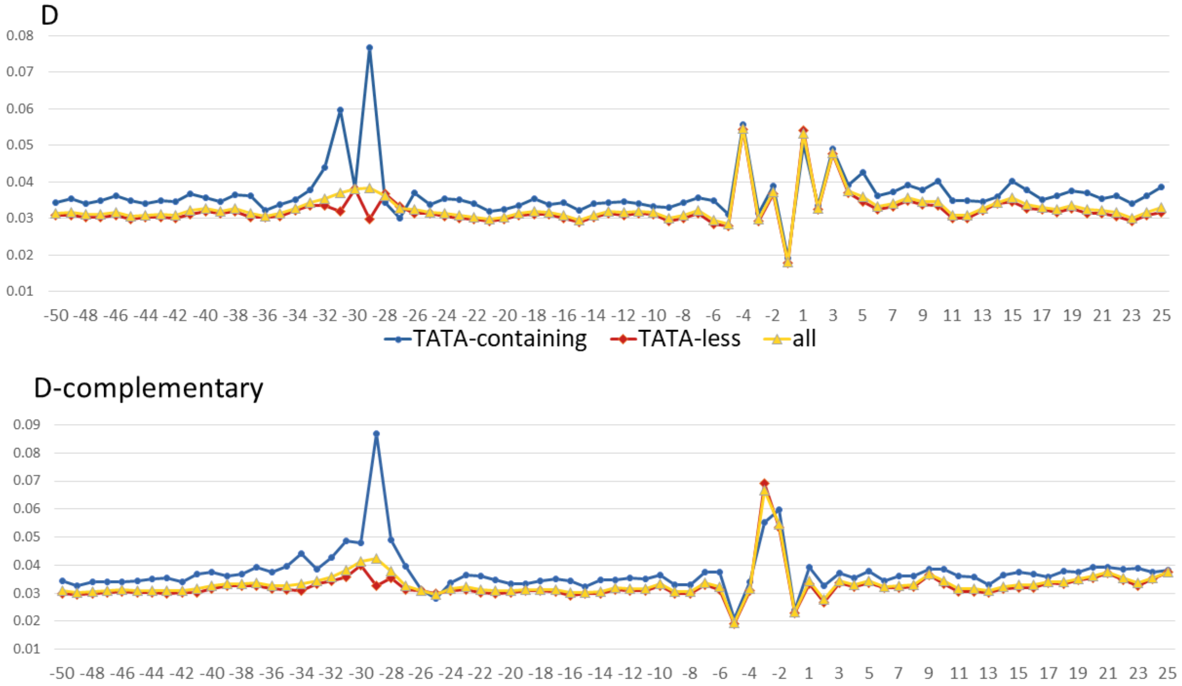


**Figure S19(a,b).** Variation of DNase I cleavage indices in core promoters of *M. musculus*.

a

b

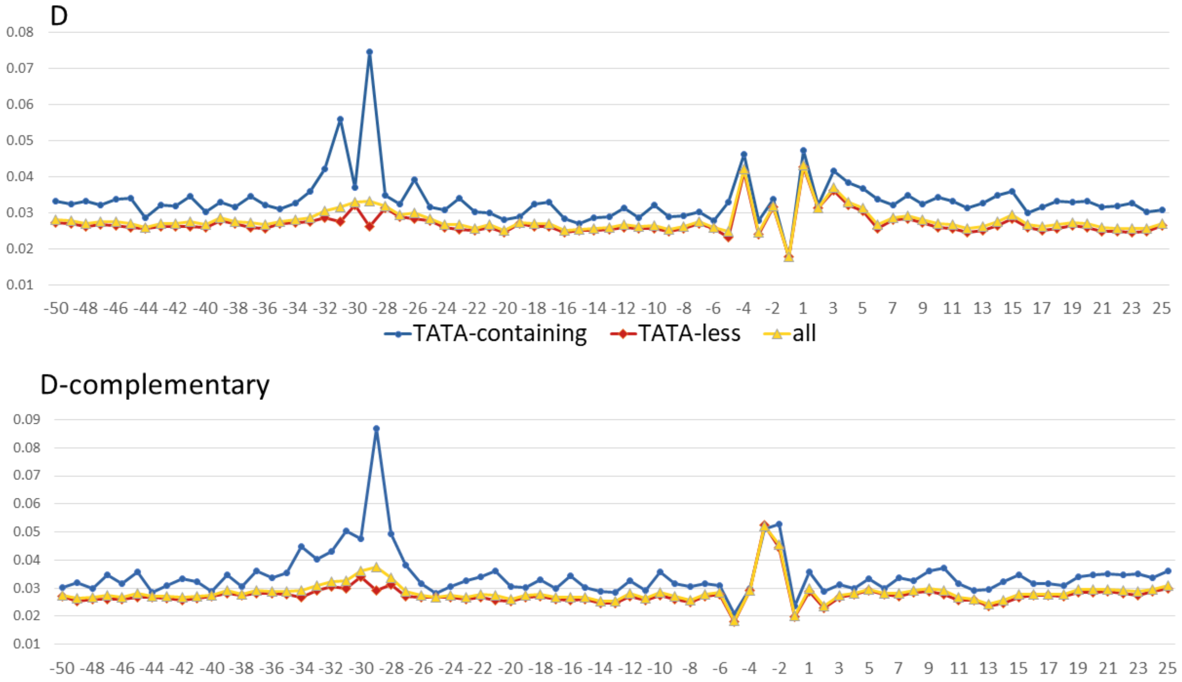


**Figure S20(a,b).** Variation of DNase I cleavage indices in core promoters of *C. familiaris*.

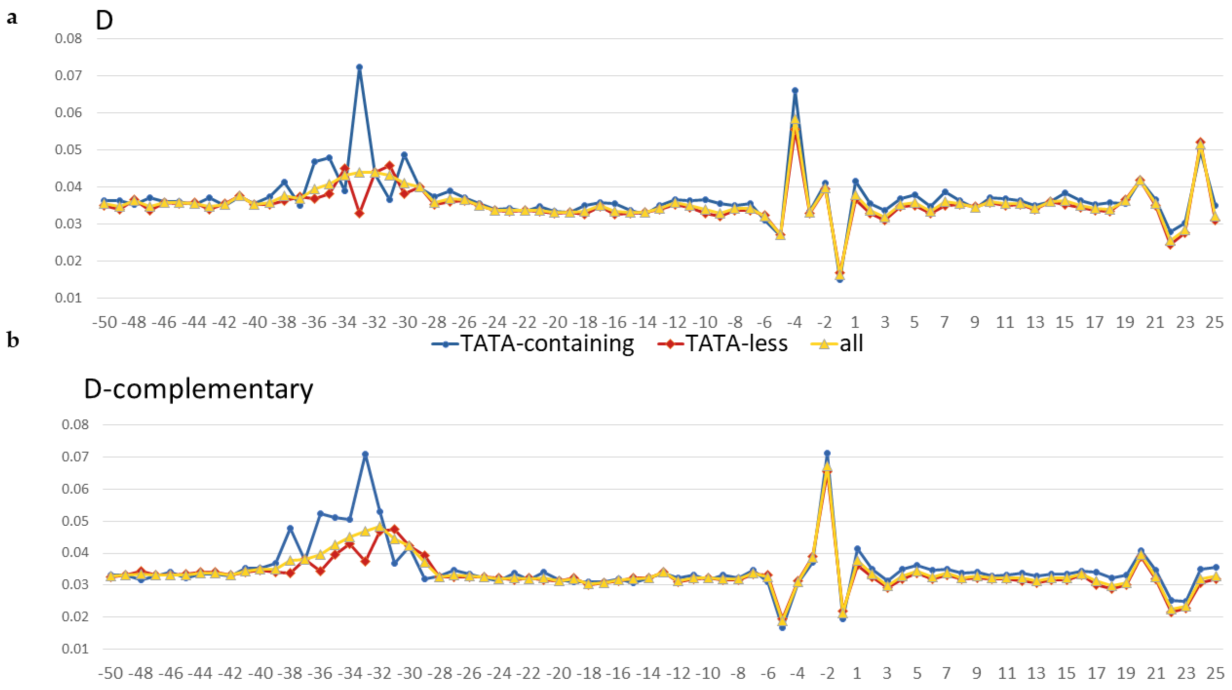


**Figure S21(a,b).**Variation of DNase I cleavage indices in core promoters of *Z. mays*.

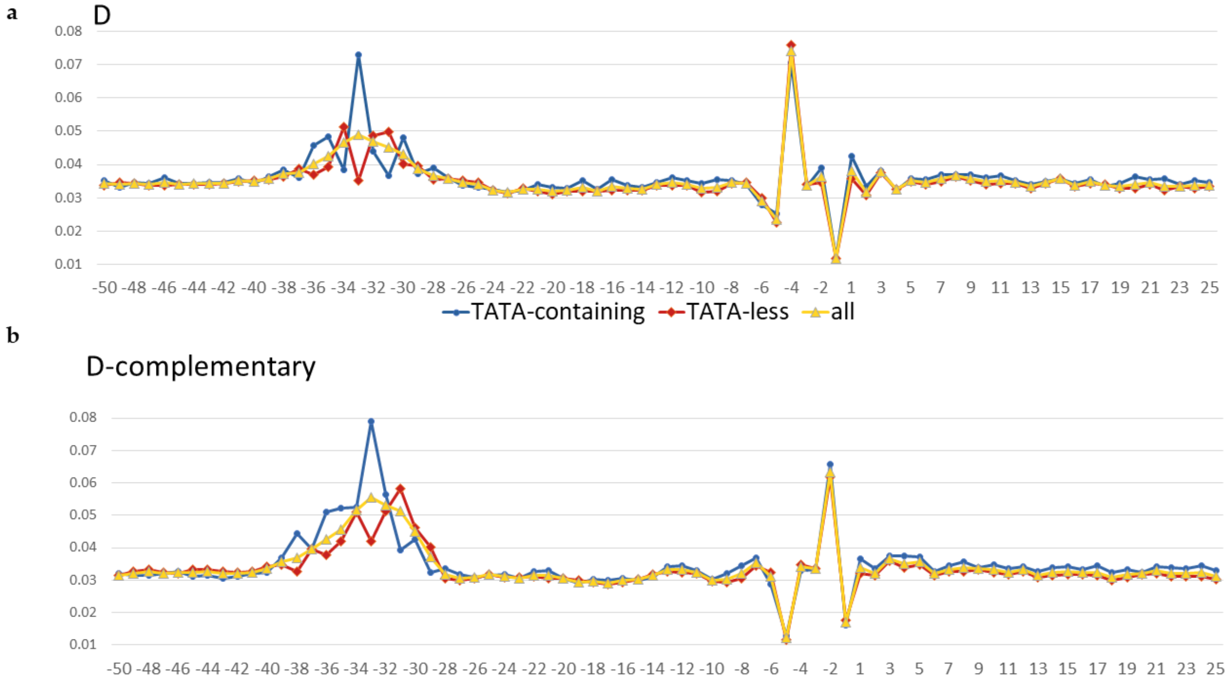


**Figure S22(a,b).** Variation of DNase I cleavage indices in core promoters of *H. vulgare*.

**Table S1.** Mann–Whitney U test results (with Bonferroni correction) comparing six structural parameters of the TATA-box octanucleotide (positions –30 to –23) between TATA-containing and TATA-less promoters of *H. sapiens*.

| dinucleotide position | Pvalue | Significantraw | Significantbonferroni | MedianTATA_containing | MedianTATA_less | Difference |
|---|---|---|---|---|---|---|
| **Mobility to bend towards the major groove** | | | | | | |
| -31:-30 | <0.000001 | True | True | 1.0800 | 1.0300 | 0.0500 |
| -30:-29 | <0.000001 | True | True | 1.0900 | 1.0300 | 0.0600 |
| -29:-28 | <0.000001 | True | True | 1.0900 | 1.0300 | 0.0600 |
| -28:-27 | <0.000001 | True | True | 1.1200 | 1.0300 | 0.0900 |
| -27:-26 | <0.000001 | True | True | 1.0900 | 1.0300 | 0.0600 |
| -26:-25 | <0.000001 | True | True | 1.0600 | 1.0300 | 0.0300 |
| -25:-24 | <0.000001 | True | True | 1.0600 | 1.0300 | 0.0300 |
| -24:-23 | <0.000001 | True | True | 1.0400 | 1.0300 | 0.0100 |
| **Stackingenergy** | | | | | | |
| -31:-30 | <0.000001 | True | True | -17.0000 | -15.8000 | -1.2000 |
| -30:-29 | <0.000001 | True | True | -17.0000 | -15.8000 | -1.2000 |
| -29:-28 | <0.000001 | True | True | -17.0000 | -15.8000 | -1.2000 |
| -28:-27 | <0.000001 | True | True | -17.5000 | -15.8000 | -1.7000 |
| -27:-26 | <0.000001 | True | True | -17.5000 | -15.8000 | -1.7000 |
| -26:-25 | <0.000001 | True | True | -16.7000 | -15.8000 | -0.9000 |
| -25:-24 | <0.000001 | True | True | -15.8000 | -15.8000 | 0.0000 |
| -24:-23 | 0.065908 | False | False | -15.8000 | -15.8000 | 0.0000 |
| **Roll** | | | | | | |
| -31:-30 | <0.000001 | True | True | 0.4000 | 1.4000 | -1.0000 |
| -30:-29 | <0.000001 | True | True | 0.4000 | 1.4000 | -1.0000 |
| -29:-28 | <0.000001 | True | True | -0.6000 | 1.4000 | -2.0000 |
| -28:-27 | <0.000001 | True | True | 1.4000 | 1.4000 | 0.0000 |
| -27:-26 | <0.000001 | True | True | 1.4000 | 1.4000 | 0.0000 |
| -26:-25 | <0.000001 | True | True | 1.4000 | 1.4000 | 0.0000 |
| -25:-24 | 0.001684 | True | False | 1.4000 | 1.4000 | 0.0000 |
| -24:-23 | 0.000200 | True | True | 1.4000 | 1.4000 | 0.0000 |
| **Rollstifness** | | | | | | |
| -31:-30 | 0.446550 | False | False | 0.0200 | 0.0190 | 0.0010 |
| -30:-29 | <0.000001 | True | True | 0.0200 | 0.0190 | 0.0010 |
| -29:-28 | <0.000001 | True | True | 0.0200 | 0.0190 | 0.0010 |
| -28:-27 | <0.000001 | True | True | 0.0200 | 0.0190 | 0.0010 |
| -27:-26 | <0.000001 | True | True | 0.0200 | 0.0190 | 0.0010 |
| -26:-25 | <0.000001 | True | True | 0.0200 | 0.0190 | 0.0010 |
| -25:-24 | <0.000001 | True | True | 0.0190 | 0.0190 | 0.0000 |
| -24:-23 | <0.000001 | True | True | 0.0190 | 0.0190 | 0.0000 |
| **Slide** | | | | | | |

| dinucleotide position | Pvalue | Significantraw | Significantbonferroni | MedianTATA_containing | MedianTATA_less | Difference |
|---|---|---|---|---|---|---|
| -31:-30 | <0.000001 | True | True | -0.1600 | 0.2800 | -0.4400 |
| -30:-29 | <0.000001 | True | True | -0.1600 | 0.2800 | -0.4400 |
| -29:-28 | <0.000001 | True | True | -0.1600 | 0.2800 | -0.4400 |
| -28:-27 | <0.000001 | True | True | -0.1600 | 0.2800 | -0.4400 |
| -27:-26 | <0.000001 | True | True | -0.1600 | 0.2800 | -0.4400 |
| -26:-25 | <0.000001 | True | True | -0.0100 | 0.2800 | -0.2900 |
| -25:-24 | <0.000001 | True | True | 0.2800 | 0.2800 | 0.0000 |
| -24:-23 | <0.000001 | True | True | 0.2800 | 0.2800 | 0.0000 |
| **Slidestifness** | | | | | | |
| -31:-30 | <0.000001 | True | True | 2.2600 | 2.0000 | 0.2600 |
| -30:-29 | <0.000001 | True | True | 2.2600 | 2.0000 | 0.2600 |
| -29:-28 | 0.000001 | True | True | 2.2600 | 2.0000 | 0.2600 |
| -28:-27 | <0.000001 | True | True | 2.2600 | 2.0000 | 0.2600 |
| -27:-26 | <0.000001 | True | True | 2.2600 | 2.0000 | 0.2600 |
| -26:-25 | <0.000001 | True | True | 2.0300 | 2.0000 | 0.0300 |
| -25:-24 | <0.000001 | True | True | 2.0300 | 2.0000 | 0.0300 |
| -24:-23 | <0.000001 | True | True | 2.0000 | 2.0000 | 0.0000 |

For each parameter in each dinucleotide position the table shows: the uncorrected p-value, significance flags before and after Bonferroni correction (raw and adjusted), median values for each group, and the median difference. Positions are given as the dinucleotide step (e.g., –31:–30 refers to the step between positions –31 and –30 relative to the TSS). The six parameters are Mobility to bend towards the major groove, Stacking energy, Roll, Roll stiffness, Slide, and Slide stiffness.

**Table S2**. Bootstrap estimates of mean differences (TATA-containing minus TATA-less) for six structural parameters of the TATA-box octanucleotide (positions –30 to –23) in *H. sapiens* promoters.

| Position | Mean | CI_lower | CI_upper | p_bootstrap | significant |
|---|---|---|---|---|---|
| **Mobility to bend towards the major groove** | | | | | |
| -31:-30 | 0.048614 | 0.047262 | 0.049843 | 0.000000 | True |
| -30:-29 | 0.060132 | 0.058812 | 0.061470 | 0.000000 | True |
| -29:-28 | 0.060461 | 0.059018 | 0.061954 | 0.000000 | True |
| -28:-27 | 0.086770 | 0.085283 | 0.088133 | 0.000000 | True |
| -27:-26 | 0.064203 | 0.062437 | 0.066049 | 0.000000 | True |
| -26:-25 | 0.039869 | 0.037871 | 0.041808 | 0.000000 | True |
| -25:-24 | 0.024029 | 0.022246 | 0.025814 | 0.000000 | True |
| -24:-23 | 0.011146 | 0.009592 | 0.012655 | 0.000000 | True |
| **Stackingenergy** | | | | | |
| -31:-30 | -0.625540 | -0.665683 | -0.586872 | 0.000000 | True |
| -30:-29 | -0.552966 | -0.583447 | -0.521086 | 0.000000 | True |
| -29:-28 | -0.770881 | -0.803775 | -0.740745 | 0.000000 | True |
| -28:-27 | -0.774324 | -0.798098 | -0.750462 | 0.000000 | True |
| -27:-26 | -0.556926 | -0.594816 | -0.515332 | 0.000000 | True |
| -26:-25 | -0.189346 | -0.237132 | -0.142320 | 0.000000 | True |
| -25:-24 | -0.159933 | -0.210088 | -0.106789 | 0.000000 | True |
| -24:-23 | -0.002874 | -0.056352 | 0.050907 | 0.469000 | False |
| **Roll** | | | | | |
| -31:-30 | -0.661904 | -0.739025 | -0.587359 | 0.000000 | True |
| -30:-29 | -1.202151 | -1.258040 | -1.143862 | 0.000000 | True |
| -29:-28 | -0.899488 | -0.967979 | -0.831875 | 0.000000 | True |
| -28:-27 | -1.075410 | -1.128218 | -1.019159 | 0.000000 | True |
| -27:-26 | -0.314165 | -0.393798 | -0.241015 | 0.000000 | True |
| -26:-25 | -0.088077 | -0.184188 | 0.010198 | 0.043000 | False |
| -25:-24 | -0.005928 | -0.115767 | 0.091807 | 0.462000 | False |
| -24:-23 | -0.069285 | -0.182144 | 0.048153 | 0.134000 | False |
| **Rollstifness** | | | | | |
| -31:-30 | -0.000510 | -0.000583 | -0.000438 | 0.000000 | True |
| -30:-29 | -0.000013 | -0.000094 | 0.000062 | 0.381000 | False |
| -29:-28 | -0.000981 | -0.001053 | -0.000907 | 0.000000 | True |
| -28:-27 | -0.000258 | -0.000321 | -0.000194 | 0.000000 | True |
| -27:-26 | -0.000283 | -0.000348 | -0.000221 | 0.000000 | True |
| -26:-25 | 0.000109 | 0.000037 | 0.000182 | 0.001000 | True |
| -25:-24 | 0.000008 | -0.000073 | 0.000084 | 0.450000 | False |
| -24:-23 | 0.000094 | 0.000012 | 0.000179 | 0.011000 | True |
| **Slide** | | | | | |

| | | | | | |
|---|---|---|---|---|---|
| -31:-30 | -0.239010 | -0.259333 | -0.218915 | 0.000000 | True |
| -30:-29 | -0.512857 | -0.525663 | -0.500545 | 0.000000 | True |
| -29:-28 | -0.184375 | -0.203019 | -0.165657 | 0.000000 | True |
| -28:-27 | -0.453891 | -0.464666 | -0.442482 | 0.000000 | True |
| -27:-26 | -0.265255 | -0.281101 | -0.247228 | 0.000000 | True |
| -26:-25 | -0.205984 | -0.224622 | -0.188943 | 0.000000 | True |
| -25:-24 | -0.105320 | -0.126531 | -0.085619 | 0.000000 | True |
| -24:-23 | -0.061592 | -0.080736 | -0.044113 | 0.000000 | True |
| **slide_stifness** | | | | | |
| -31:-30 | 0.148284 | 0.124214 | 0.173787 | 0.000000 | True |
| -30:-29 | 0.317717 | 0.290306 | 0.347768 | 0.000000 | True |
| -29:-28 | 0.034031 | 0.007386 | 0.059711 | 0.003000 | True |
| -28:-27 | 0.192059 | 0.168450 | 0.214986 | 0.000000 | True |
| -27:-26 | 0.111758 | 0.091927 | 0.132886 | 0.000000 | True |
| -26:-25 | 0.124059 | 0.105934 | 0.143162 | 0.000000 | True |
| -25:-24 | 0.077479 | 0.059838 | 0.097865 | 0.000000 | True |
| -24:-23 | 0.038285 | 0.022872 | 0.054221 | 0.000000 | True |

For each parameter in each dinucleotide position the table shows: the mean difference (Δ), the 95% confidence interval (percentile method, 1,000 bootstrap iterations), an approximate p-value (proportion of bootstrap replicates where the difference had the opposite sign), and a significance flag (True if the 95% CI does not contain zero). Positions are denoted as dinucleotide steps relative to the TSS. The six parameters are Mobility to bend towards the major groove, Stacking energy, Roll, Roll stiffness, Slide, and Slide stiffness.

* diff_mean — the difference in means between groups (TATA-containing minus TATA-non-containing).

CI_lower / CI_upper — the 95% confidence interval for this difference.

p_bootstrap — the approximate p-value (the proportion of bootstrap differences with a sign opposite to the observed one).

significant = True if the confidence interval does not contain 0 (i.e., the difference is statistically significant at the 5% level).
